# Identifying dementia neuropathology using low‐burden clinical data

**DOI:** 10.1002/alz.70539

**Published:** 2025-08-04

**Authors:** Yueqi Ren, Babak Shahbaba, Craig E. L. Stark

**Affiliations:** ^1^ Medical Scientist Training Program School of Medicine University of California Irvine Irvine California USA; ^2^ Department of Statistics Donald Bren School of Information and Computer Sciences University of California Irvine Irvine California USA; ^3^ Department of Neurobiology and Behavior University of California Irvine Irvine California USA

**Keywords:** Alzheimer's disease, clustering, diagnosis prediction, mixed dementia, neuropathology screening, progression monitoring, semi‐supervised learning, statistical machine learning

## Abstract

**INTRODUCTION:**

Identifying dementia neuropathology is critical for guiding effective therapies and clinical trials. To tackle this, we developed semi‐supervised models for identifying neuropathology using low‐burden data to improve generalizability.

**METHODS:**

We defined low‐burden data as being reasonably obtainable at a primary care setting. By using a semi‐supervised learning paradigm, we can amplify the utility of low‐burden data. We trained a clustering and a semi‐supervised prediction model to yield clustering and prediction results for different neuropathology lesion types.

**RESULTS:**

Our clustering model identified two clinically meaningful outlier groups that were either neuropathology‐enriched or ‐scarce. We predicted neuropathology burden across different pathology types and found that using low‐burden data over multiple clinical visits can predict neuropathology on par with using higher‐burden data.

**DISCUSSION:**

This work fills a critical gap in the field by using low‐burden clinical data to predict neuropathology, thereby improving dementia screening, therapy, and targeted clinical trials.

**Highlights:**

- Clinical data are useful for neuropathology screening in future clinical trials.
- Novel application of semi‐supervised learning for identifying neuropathology.
- Clustering model found groups with highly different neuropathology prevalence.
- Low‐burden data can provide relatively accurate predictions of pathology load.
- Higher‐burden, longitudinal data are most helpful for predicting vascular lesions.

## 1 BACKGROUND

Effective screening for Alzheimer's disease (AD) and related dementias is a major bottleneck for early detection.^1^ A majority of dementia cases are first diagnosed by non‐specialist healthcare providers.^2^, ^3^, ^4^ Therefore, collaborative primary care models are critical for diagnosis and treatment.^5^, ^6^ Few sensitive biomarkers are used clinically, partly due to lack of longitudinal and reproducible follow‐up.^7^ Currently, only the recently approved pTau217/ß‐amyloid 1‐42 plasma ratio for AD amyloid plaque detection is available. We also have limited understanding of how to best utilize existing, low‐burden clinical data, such as demographics, health history, and behavioral survey data, for disease screening across pathologies. Most studies rely upon higher cost information, such as the Clinical Dementia Rating (CDR) used in clinical trials and research centers, for diagnosis prediction.^8^, ^9^, ^10^, ^11^ Some have demonstrated accurate predictive performance using low‐burden data for diagnoses of mild cognitive impairment (MCI) and dementia as well as monitoring progression.^12^, ^13^ Few, important studies have extended past clinical diagnosis to predict disease etiology using clinical data.^14^, ^15^ None have focused on using low‐burden data to predict a wide range of dementia neuropathology, including trans‐active response DNA‐binding protein of 43 kDa (TDP‐43) and Lewy body disease. Predicting coexisting neuropathology is especially critical given that co‐pathologies are more common and may precipitate greater cognitive decline.^16^ As the field moves to biological staging of AD,^17^ a critical gap is understanding how well low‐burden data can identify neuropathology burden.

**RESEARCH IN CONTEXT**

1. **Systematic review**: Using PubMed, the authors conducted a literature review for predicting dementia neuropathology using low‐burden clinical data (search terms included Alzheimer's/dementia/neurodegeneration, machine learning/artificial intelligence/prediction, and neuropathology/autopsy/lesion). No studies have exclusively used low‐burden clinical data to predict neuropathology, and most biomarker studies had limited scope, focusing on either a small set of biomarkers or a specific neuropathology. There is a critical gap in knowledge in screening for dementia neuropathology using low‐burden clinical data.
2. **Interpretation**: We developed a framework for identifying and predicting neuropathology using semi‐supervised methods. This included a clustering model that learns from individuals without neuropathology data available prior to identifying meaningful subgroups of individuals with neuropathology data. Additionally, we developed a semi‐supervised model to predict neuropathology burden and demonstrated robust prediction performance using low‐burden data.
3. **Future directions**: Low‐burden clinical data can effectively detect dementia neuropathology and should be considered as screening tools in clinical trials.

While AD biomarker research has made impressive strides, autopsy‐confirmed neuropathology remains the gold standard for diagnosing AD and other dementias.^18^ In this area, the applications of low‐burden clinical data (Table 1) have been underexplored. Past studies almost exclusively focused on highly sensitive biomarkers, mainly neuroimaging, and include only a single or small set of neuropathology.^14^, ^15^, ^19^, ^20^ While these studies demonstrated that clinical data and neuroimaging correlate well with neuropathology, especially AD‐ and amyloid‐related lesions, key knowledge gaps remain.^15^, ^21^, ^22^ In major community‐based studies, most older adults with neurocognitive impairments have mixed neuropathology, but it is unclear how well clinical data can predict cumulative neuropathology.^23^, ^24^ Additionally, most of these higher‐burden biomarkers remain out of reach for patients. This is especially true for people facing health disparities who typically experience greater dementia prevalence, burden of cognitive impairment, and costs of caregiving.^25^ Rural populations with lower socioeconomic status face greater challenges accessing care.^26^ Research advances also typically disproportionately benefit more economically privileged and highly educated persons.^25^, ^27^ Taken together, these concerns point toward the importance of providing accessible neuropathology screening that only uses low‐burden data.

**TABLE 1:** Features stratified by cost and burden.

Tier	Modality	Features
1: Low‐burden	Demographics	Age, sex, education level
		Race, ethnicity, language(s) spoken
		Marital status, living situation
	Patient history	Tobacco use, alcohol use, medications
		Cardiovascular conditions and comorbidities (e.g., congestive heart failure, stroke, diabetes)
		Family history of dementia
		Physical exam (e.g., heart rate, blood pressure, BMI)
	Behavioral surveys	NACC Functional Assessment Scale
		Neuropsychiatric Inventory Questionnaire
		Geriatric Depression Scale
	Neuropsychological testing	Mini‐Mental State Exam
2: Medium‐burden	Neuropsychological testing	Logical Memory II—Delayed, Trails A and B, Boston Naming Test, vegetable and animal naming, digit span
3: High‐burden	Genetic testing	ApoE allele carrier status
	Clinical Dementia Rating	CDR global, CDR sum of boxes, subdomain scores

Abbreviations: ApoE, apolipoprotein E; BMI, body mass index; NACC, National Alzheimer's Coordinating Center.

Here, we seek to evaluate the utility of low‐burden clinical data in identifying a range of dementia neuropathology. We define low‐burden data as information that does not require substantial clinical cost, time, or expertise to administer (see Table 1). These features could be reasonably obtained by a general practitioner, non‐physician provider, or caregiver. We compare these features against medium‐ and high‐burden information that is typically obtained only by specialty providers or in research settings. Past work established that low‐burden data can predict AD clinical diagnoses comparably to neurologists and significantly better than primary care screening.^13^ Building from this, our first goal is to improve clinical trial screening by applying statistical machine learning to identify meaningful subgroups with differing neuropathology risk. Our second goal is to provide informative neuropathology diagnostics using low‐burden data. We hypothesize that using statistical machine learning will yield more precise neuropathology predictions than previously obtained with clinical diagnosis.^28^ We also hypothesize that using longitudinal, low burden data can achieve comparable predictions as using cross‐sectional, high burden data. For vascular pathologies, we hypothesize that using higher burden data will significantly boost prediction performance due to low‐burden data's limited predictive power.^29^ We evaluated various machine learning methods, especially semi‐supervised models, to evaluate the efficacy of different types of data in predicting co‐existing neuropathology. We also identified subgroups with significantly lower and higher prevalence of neuropathology. This study is one of the first to determine the clinical utility of low‐burden data in identifying neuropathology.

## 2 METHODS

### 2.1 Data description

Data used in this study come from the National Alzheimer's Coordinating Center (NACC) and includes the Uniform Data Set (UDS) and Neuropathology (NP) Data Set.^30^ The UDS contains clinical information, behavioral survey responses, neuropsychological testing results, and additional diagnostic information for each subject.^31^, ^32^, ^33^ The NP Data Set contains detailed pathologic lesion information collected in a similar manner across sites and includes more than 90 parameters across various domains of pathology.^34^ All participants have UDS data, and a subset of participants who underwent autopsy and neuropathology evaluation have NP data (Table S1).

**FIGURE 1:**
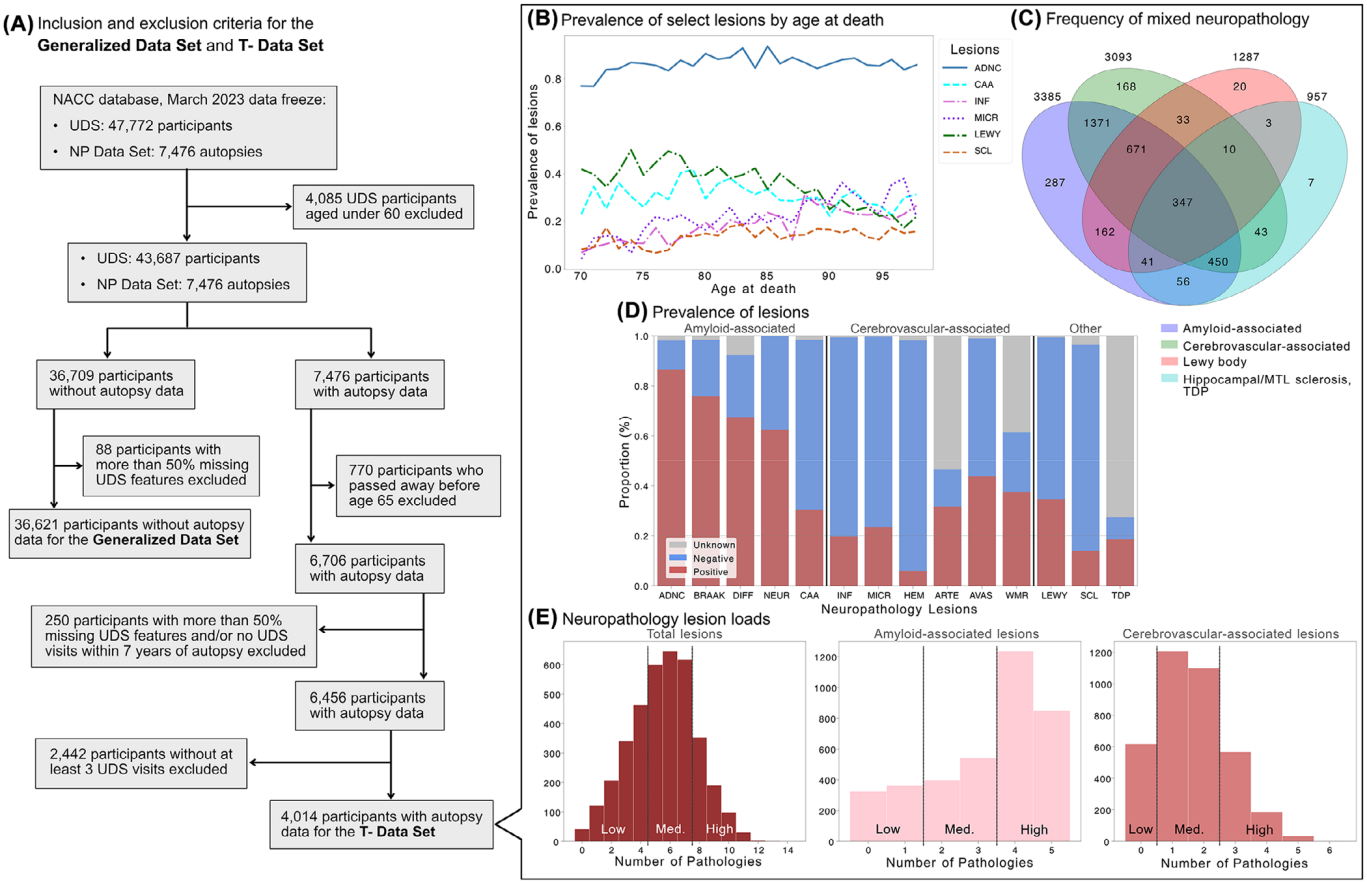
Generation of datasets and description of neuropathology lesions. (A) Summary of the inclusion and exclusion criteria used to create the Generalized Data Set, which includes all subjects without autopsy data over the age of 60 years, and the T‐ Data Set, which includes all subjects with autopsy obtained at age 70 or older with longitudinal data. B: Prevalence of select neuropathology lesions reveals relatively consistent lesion prevalence across different ages at death. Some cerebrovascular lesions have greater prevalence over time while Lewy body disease showed lower prevalence across years lived. C: Mixed dementia neuropathology is very common in our dataset and most patients have more than one type of neuropathology. D: Cross‐sectional prevalence of all neuropathology lesions shows high prevalence of AD and amyloid‐associated lesions. Prevalence of cerebrovascular‐associated lesions vary greatly, with few patients having hemorrhages and microbleeds and many having atherosclerosis of the circle of Willis. E: For each type of neuropathology, we quantified the pathology load and identified three levels of load burden for prediction labels. Amyloid‐associated lesions include ADNC (Alzheimer's disease neuropathologic change), BRAAK (Braak stage), NEUR (density of neocortical neuritic plaques), DIFF (density of diffuse plaques), CAA (cerebral amyloid angiopathy); cerebrovascular‐associated lesions include INF (infarcts), MICR (microinfarcts), HEM (hemorrhages and microbleeds), ARTE (arteriolosclerosis), AVAS (atherosclerosis of the circle of Willis), WMR (white matter rarefaction); Other lesions include LEWY (Lewy body disease), SCL (hippocampal and/or medial temporal lobe sclerosis), TDP (trans‐active response DNA‐binding protein of 43 kDa).

We outline several data inclusion and exclusion criteria here, summarized in Figure 1A. Because the goals of our study focus on sporadic forms of neurodegeneration, we excluded any participants under the age of 60 years for all analyses. We then created two analysis‐specific datasets using the UDS and NP data. To investigate whether clinically meaningful distinctions translated into meaningful differences in neuropathology, we define a *Generalized Data Set* that included all participants who have not undergone neuropathology assessment at autopsy. This dataset included 36,621 participants each with one clinical visit that was randomly selected across all available visits to obtain a representative sample of the clinical landscape across age and clinical status. Next, to investigate the effects of longitudinal clinical information on understanding neuropathology risk, we defined a *T‐ Data Set* (“time minus”) that included all participants who have undergone neuropathology assessment at autopsy (Figure S1). This dataset included longitudinal clinical visits spaced approximately a year apart starting from the date of autopsy, hence the name “time minus (T‐)”. We excluded any participants who underwent autopsy before the age of 65 years to focus the analysis on older participants who may have more sporadic forms of neuropathology accumulate over time (Figure 1B‐D), with additional inclusion and exclusion details in Supplemental Materials. This dataset included 4,014 participants with varying number of clinical visits, from three to seven UDS visits.

From the NP Data Set, we identified a subset of the most meaningful neuropathology lesions to analyze using both past work and sample limitations. This set included 14 total lesions that span across a few major domains of pathology, including amyloid‐associated pathologies, cerebrovascular‐associated pathologies, Lewy body disease, TDP‐associated pathologies, and hippocampal/medial temporal lobe (MTL) sclerosis (Figure 1D). Because Braak staging in this dataset does not serve as a mediator for amyloid plaque pathology's effect on cognitive changes, which was tested using mediation analyses, we included Braak staging within the other amyloid‐associated pathologies due to its contribution to the development of AD and potential influence on amyloid pathology development. Definitions for the neuropathology lesions and binarized labels are listed in Table 2, with a breakdown of the prevalence of each neuropathology included in Table S1 and additional methodological details in Supplemental Materials.

**TABLE 2:** Neuropathology lesion binarization.

Pathology type	Variable	Definition	Binarization (low / high)
Amyloid‐associated	ADNC	Alzheimer's disease neuropathologic change	Absent / present
	BRAAK	Braak stage	None, I‐II / III‐VI
	DIFF	Density of diffuse plaques	None, sparse / inter., freq.
	NEUR	Density of neocortical neuritic plaques	None, sparse / inter., freq.
	CAA	Cerebral amyloid angiopathy	None, mild / moderate, severe
Cerebrovascular‐associated	INF	Infarcts	Absent / present
	MICR	Microinfarcts	Absent / present
	HEM	Hemorrhages and microbleeds	Absent / present
	ARTE	Arteriolosclerosis	None, mild / moderate, severe
	AVAS	Atherosclerosis of the circle of Willis	None, mild / moderate, severe
	WMR	White matter rarefaction	None, mild / moderate, severe
Other	LEWY	Lewy body disease	Absent / present
	SCL	Hippocampal and/or medial temporal lobe sclerosis	Absent / present
	TDP	pTDP‐43 across 5 regions	Absent / present

Past work has shown that neurodegenerative processes have summative, deleterious effects on cognition and brain health, which can be captured by measuring the total load of pathology in the brain.^35^ This notion also captures mixed dementia pathologies that are prevalent in the older adult population,^36^, ^37^ as evidenced by the high number of participants with multiple types of lesions in Figure 1C. We quantified the level of neuropathology load by calculating the number of lesions present for all pathology types, for amyloid‐associated pathologies only, and for cerebrovascular‐associated pathologies only. By assessing the distribution of neuropathology load for each pathology domain, we defined cutoffs to identify none/low, medium, and high levels of pathology load to enable more feasible multiclass prediction of neuropathology burden. More details can be found in the Supplemental Materials.

### 2.2 Feature tiers stratified by cost and implementation burden

To investigate the utility of low‐burden clinical data, we stratified clinical features into tiers based upon their level of accessibility, cost, and burden in line with our previous work.^13^ Tier 1 features are low‐burden and could be obtained at low costs measured as clinical time, healthcare costs, resources needed, and burden of administration. Tier 2 features are medium‐burden and are typically only obtainable at specialty care settings. In the UDS, this corresponded to features from a full battery of neuropsychological testing. Tier 3 features are high‐burden and only obtained for research studies or clinical trials. Here, this included scores from the CDR and genetic testing for apolipoprotein E (ApoE) genotyping. We illustrate these tiers in Table 1 with additional details in Supplemental Materials. We assessed the availability of fluid biomarkers and neuroimaging data for this sample and found that requiring subjects to have additional modalities of data would significantly reduce our sample size. As more fluid biomarker data becomes available, future work incorporating these biomarkers into similar analyses will be a necessary next step.

### 2.3 Data processing

To ensure that all features of interest were adequately represented in our sample, we performed several feature processing steps, including removing features with excessive missingness and scaling features appropriately (all UDS features used included in Table S2). For the T‐ Data Set, we further extracted longitudinally derived features from subjects with at least three UDS visits. Doing so allowed us to gain longitudinal information regarding trends and variability of clinical data over time without relying on models specific to longitudinal events (e.g., recurrent neural networks), which can be noisier given the irregular temporal pattern of visits and variability of number of visits across subjects, to prevent over‐ or underfitting due to our limited sample size and number of visits. Processing details and features used are included in Supplemental Materials.

### 2.4 Clustering analysis to identify enriched subgroups

Our first goal is to use only low‐ or medium‐burden clinical information to identify subgroups that have significantly enriched neuropathology lesions compared to chance. We used the Generalized Data Set for the clustering analyses, which does not include any neuropathology data, to identify representative clusters across the entire clinical landscape using either tier 1 or tiers 1–2 features. After exploring a range of techniques, we build a pipeline using dimensionality reduction based upon a priori features followed by an autoencoder and a Gaussian mixture model (GMM). Further details are included in Supplemental Materials. We then ranked the clusters from least to most clinically impaired based on the proportion of participants without any level of cognitive impaired as diagnosed by clinicians.

After fitting the tuned GMM on the Generalized Data Set, we assigned participants with autopsy data from the T‐ Data Set to the three predefined clusters at each available clinical timepoint prior to death. Prior to subsequent analyses, we identified three different possible cluster assignment trajectories over time. Group C trajectories included participants who began in a less impaired cluster and moved to a more impaired cluster over time. Group B trajectories included participants who, on average, did not change cluster assignments over time. Group A trajectories included participants who began in a more impaired cluster and moved to a less impaired cluster over time. Due to volunteer bias in clinical visits occurring 1‐2 years before death, and our aim of using earlier time points to infer neuropathology for therapeutic translation, we excluded visits 1‐2 years before death when defining the trajectory subgroups. For all participants with autopsy, we calculated the proportion of positive lesions for each neuropathology, stratified by cluster trajectory type (groups A, B, C). To evaluate our results, we computed a null distribution of the proportion of positive lesions across all participants for each time point using nonparametric bootstrapping.^38^ We randomly sampled subjects with replacement 30,000 times to obtain a distribution of estimates of the true proportion of positive lesions. Because the estimates can be biased, we used a bias corrected and accelerated bootstrap to correct for both bias and skewness in the bootstrap distribution.^39^, ^40^ We calculated the 99.9% confidence interval to account for potential underestimation of the true confidence interval and multiple comparisons. Proportions of positive lesions that fell outside of the confidence interval were reported as significant.

### 2.5 Classification pipeline to predict neuropathology status

Our second goal is to make predictions of neuropathology lesion positivity using only limited features from tier 1, tier 1–2, or tier 1–3 with and without longitudinal clinical information. We compared three aspects of the classification pipeline: the prediction models, the feature tiers, and the presence of longitudinal clinical information. For the prediction models, we compared supervised machine learning methods, including logistic regression, random forest, and gradient boosting, against semi‐supervised methods. We found that all three of the more complex models (random forest, gradient boosting, semi‐supervised learning) yielded comparable results across all analyses and performed significantly better than the benchmark logistic regression model. Semi‐supervised machine learning enables us to overcome sample size limitations of obtaining autopsy neuropathology assessments and reduce the bias associated with obtaining brain donations for the NP Data Set by leveraging information from participants without autopsy. We derived longitudinal clinical information using participants from the T‐ Data Set with three or more clinical visits as outlined above. To contrast against the longitudinal information, we also obtained cross‐sectional information for a second dataset by randomly sampling a clinical visit for each subject with autopsy, similar to the Generalized Data Set, to determine if longitudinal information improves predictions of neuropathology. We then developed a semi‐supervised deep generative model using a variational autoencoder and deep neural network to predict neuropathology. Modeling details can be found in Supplemental Materials.

### 2.6 Computing feature importance for low‐burden data

To investigate the contributions of low‐burden data in predicting neuropathology, feature importance using leave‐one‐covariate‐out importance was computed for each classification task.^41^ We compared these results with permutation feature importance and found similar importance trends. Features with significant importance values have 95% confidence intervals that lie above the chance line at zero (no change in feature importance). Remove of these features would result in a decline in the prediction area under the receiver operating characteristic curve (ROC‐AUC) and produce a negative relative change in ROC‐AUC (normalized by the original model's ROC‐AUC).

## 3 RESULTS

### 3.1 Relationships between neuropathology lesions

We characterized the relationships between different types of neuropathology lesions, as mixed dementia pathologies are common (Figure 1C). To assess mixed dementia pathologies, we defined three labels for total neuropathology load, amyloid‐associated neuropathology load, and cerebrovascular‐associated neuropathology load (Figure 1E). When assessing the relationships between each of the load types, we found that amyloid‐associated load contributed the most to the total neuropathology load in each patient and has a relatively linear relationship with total load (Figure S2A). In contrast, cerebrovascular‐associated lesions has a more nonlinear relationship with total neuropathology load, thus contributing less to the overall pathology burden. When comparing amyloid‐ and cerebrovascular‐associated load, we observed that while amyloid‐associated lesions increased, cerebrovascular‐associated lesions did not change substantially, staying relatively concentrated around 1–2 lesions. These trends remained consistent after binning each of the load types into low, medium, and high groups (Figure S2B). Our findings suggest that amyloid‐ and cerebrovascular‐associated lesions do not accumulate in a dependent manner and amyloid‐associated pathologies make up most of the neuropathology found present postmortem.

**FIGURE 2:**
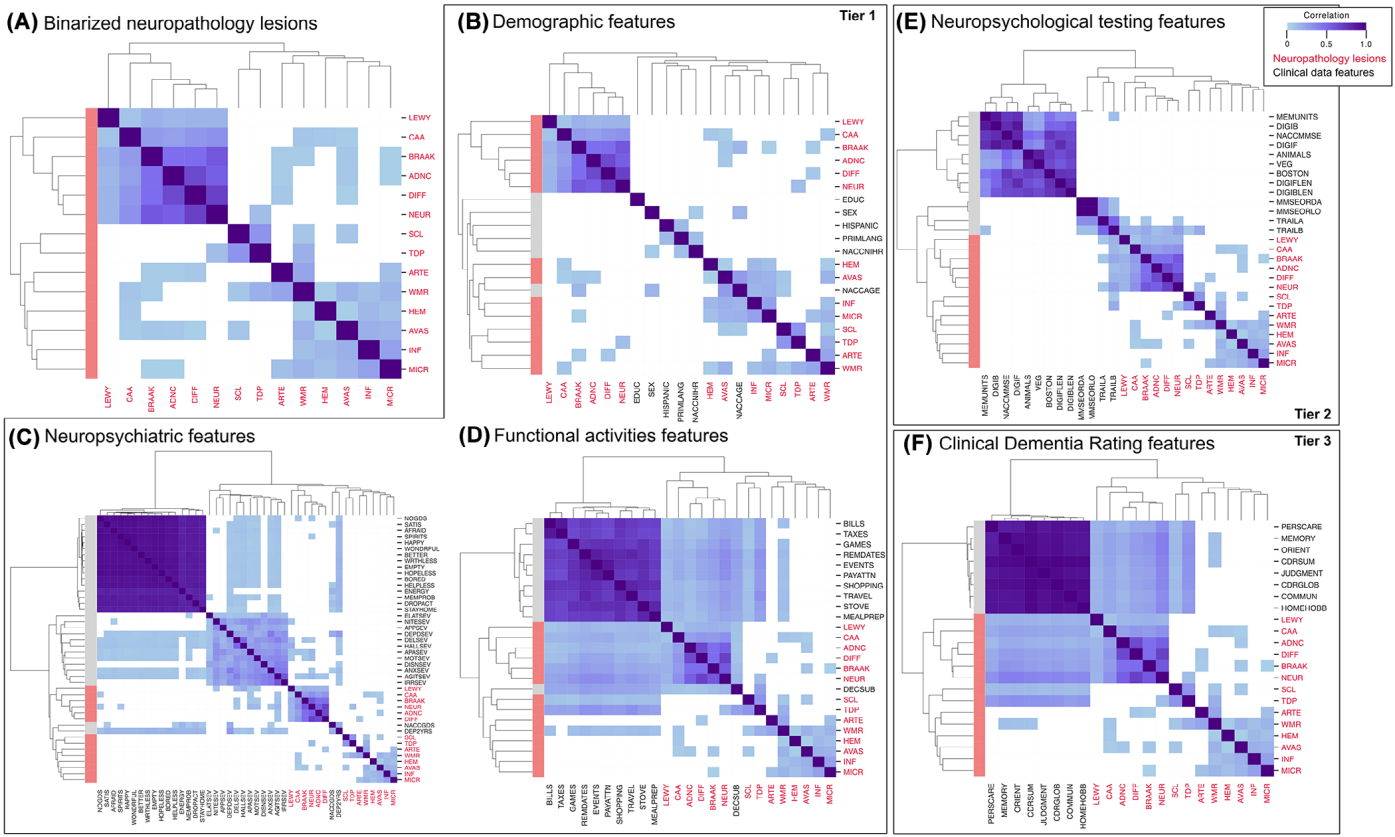
Correlations between key feature modalities and neuropathology lesions showed limited signals for most modalities. Overall, A shows the correlation among neuropathology lesions, B‐D are correlations between tier 1 features and neuropathology, E highlights correlation between tier 2 neuropsychological features and neuropathology, and F shows the correlation between tier 3 CDR features and neuropathology. A: Two main clusters were found among neuropathology lesions, an amyloid‐associated cluster and a cerebrovascular‐associated cluster. B: Demographic features, in general, showed very few significant relationships with neuropathology, apart from age. C: Some neuropsychiatric and depression features were significantly correlated with amyloid‐associated neuropathology lesions. D: Functional activities features were significantly associated with non‐cerebrovascular lesions, excluding WMR. E: Trails A and B were significantly associated with non‐cerebrovascular lesions. In addition, both features alongside with the orientation sub‐scores from the MMSE were clustered with neuropathology. B: CDR features were significantly associated with non‐cerebrovascular lesions, similar to the relationship for functional activities features. Pearson correlation coefficients with *p*‐values < 0.05 after Bonferroni correction were plotted. CDR, Clinical Dementia Rating; WMR, white matter rarefaction.

Among neuropathology lesions, we identified two major clusters, a prominent amyloid‐associated cluster, which also included Lewy body disease at the edge of the cluster, and a less prominent cerebrovascular‐associated cluster, which included hippocampal/MTL sclerosis and TDP‐43 pathologies at the periphery (Figure 2A, color threshold based on Bonferroni‐corrected *p*‐value for the Pearson correlation coefficient). These findings corroborated our earlier observations. Sclerosis of the hippocampus and/or MTL and high density of neuritic plaques were significantly associated with TDP‐43 pathology, while Lewy body disease was not associated with CAA. A few cerebrovascular lesions, including white matter rarefaction, atherosclerosis, and arteriolosclerosis, were significantly correlated with amyloid‐associated lesions albeit with small correlation coefficients.

### 3.2 Associations between neuropathology and clinical features

Prior to predictive modeling, we investigated correlations between modality‐specific sources of clinical information and neuropathology lesions. Between neuropathology and tier 1 clinical domains, we identified only a few significant correlations, suggesting that more advanced machine models are needed to improve prediction performance. Among demographic features, only age was significantly associated with neuropathology, specifically high Braak stage and some cerebrovascular lesions (Figure 2B). For neuropsychiatric features, there were prominent clusters of items from the Geriatric Depression Scale (GDS) and Neuropsychiatric Inventory Questionnaire (NPI‐Q). Among these, only the total GDS score (NACCGDS) and having active depression within the last 2 years (DEP2YRS) were clustered with cerebrovascular‐associated lesions, but correlations were not significant after Bonferroni correction. Among NPI‐Q features, severity of agitation/aggression (AGITSEV), apathy/indifference (APASEV), motor disturbance (MOTSEV), and delusions (DELSEV) were significantly correlated with amyloid‐associated lesions, and some with Lewy body disease. Severity of hallucinations (HALLSEV) was significantly correlated with Lewy body disease as well (Figure 2C). For functional activities features, which overlap with living situation and CDR features, we observed significant correlations between many items of the Functional Assessment Scale (FAS) and amyloid‐associated lesions, TDP‐43, white matter rarefaction, and sclerosis of the hippocampus/MTL. The latter two lesions had weaker, but still significant correlations with FAS features. Notably, the participant's subjective report of cognitive decline (DECSUB) was the only feature to be clustered with the neuropathology lesions and was strongly associated with TDP‐43 pathology (Figure 2D). While this association is significant, due to the small sample size of individuals with autopsy‐confirmed TDP‐43 pathology, care must be taken to interpret this finding within the context of this dataset. Other notable associations include family history features, including ApoE genotype, with amyloid‐associated lesions but not with cerebrovascular‐associated lesions; physical exam findings, especially hearing, with amyloid‐associated lesions; living situation features with TDP‐43 and neuritic plaques, especially, in addition to other amyloid‐associated lesions (Figure S3). Details regarding these tier 1 modality associations are further described in Supplemental Materials.

In addition to the above tier 1 feature domains, we also assessed the relationship between tier 2 and tier 3 features and neuropathology. For neuropsychological testing features, we found that the Trail Making Test Parts A & B, especially Trails B, were significantly correlated with amyloid‐associated lesions, TDP‐43, Lewy body disease, and hippocampal/MTL sclerosis (Figure 2E). ApoE e4 allele carriership status was significantly correlated with amyloid‐associated lesions only (Figure S3A). For CDR features, we observed significant correlations between the total scores and domain scores with amyloid‐associated lesions, TDP‐43, Lewy body disease, hippocampal/MTL sclerosis, and white matter rarefaction (Figure 2F). The correlation was especially strong for neuritic plaque and TDP‐43 pathology, echoing the trend found in tier 1 features.

In summary, among tier 1 features, neuropsychiatric and functional activities domains had the most notable associations with neuropathology. Among tier 2 features, Trails B was strongly associated with several lesions. All features in tier 3, including ApoE and CDR scores, were significantly associated mostly with non‐cerebrovascular type lesions. While these significant associations exist, most clinical features do not strongly correlate with neuropathology, providing evidence that more sophisticated, data‐driven methods, such as machine learning models, are needed to link the two types of data.

### 3.3 Identifying enriched subgroups for neuropathology using limited clinical features from participants without autopsy

**FIGURE 3:**
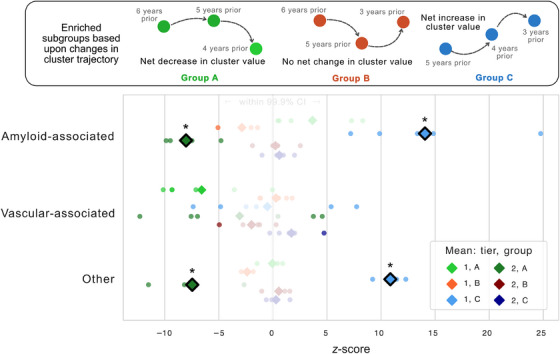
Representative clustering analysis identified two outlier groups with significantly different prevalence of neuropathology lesions. We identified three cluster assignment trajectories that were likely to be associated with differing risks of decline over time (inset). The proportion of neuropathology lesions within each group was reported using *z*‐scores compared to a null distribution across the entire study population. Group A generally had lower prevalence of neuropathology, which was statistically significant using tier 1‐2 features for amyloid‐associated lesions (ADNC, Braak stage, density of diffuse and neuritic plaques, and CAA) and other, non‐cerebrovascular lesions (TDP‐43, and sclerosis of the hippocampus and/or MTL). Group B tended to have *z*‐scores around the null distribution (lower opacity indicates lack of statistical significance). Group C had consistently greater prevalence of various neuropathology lesions, which was significant using tier 1 features for all amyloid‐associated and other, non‐cerebrovascular lesions (bolded diamond indicates significant mean *z*‐score). ADNC, Alzheimer's disease neuropathologic change; CAA, cerebral amyloid angiopathy; MTL, medial temporal lobe; TDP‐43, trans‐active response DNA‐binding protein of 43 kDa.

For the general aging population, we often cannot collect extensive biomarker information beyond widely accessible, low‐burden clinical information. Given this, we prioritized using widely accessible, lower‐cost features in our clustering algorithm to identify potentially meaningful subgroups that reveal hidden information about latent neuropathologic lesions. After obtaining clusters using either tier 1 or tiers 1–2 features from participants without autopsy information and ranking clusters by the severity of clinical diagnoses, we defined three possible cluster assignment trajectories (see Figure 3, inset; Figure S4B). Group A has a trajectory of decreasing cluster assignment/value (i.e., going from a cluster with more severe clinical diagnoses on average to one with less severe diagnoses), which we predict is likely associated with a lower risk of decline over time compared to their counterparts. Group B has no net change in cluster value (a “flat” trajectory when comparing the first and final time points), and group C has a net increase in cluster value (i.e., going from a less clinically severe to a more severe cluster), which we predict is likely associated with a greater risk of decline over time.

We then investigated whether neuropathology lesion prevalence differed between these groups (A–C) compared to an expected, null distribution across the entire study population. Across all lesion types, we discovered two outlier subgroups of individuals that had either significantly lower or higher prevalence of amyloid‐associated and other, non‐cerebrovascular lesions. We consistently observed that group A had lower prevalence of neuropathology. This trend was statistically significant compared to a 99.9% confidence interval from the null distribution when using tier 1–2 features for clustering in the proportion of amyloid‐associated lesions (ADNC, Braak stage, density of diffuse and neuritic plaques, and CAA) and other, non‐cerebrovascular lesions (TDP‐43, and sclerosis of the hippocampus and/or MTL). While some cerebrovascular‐associated lesions had significantly lower prevalence in group A, the average *z*‐score of these lesions was not significantly different from the null (Figure 3, lower opacity indicates lack of statistical significance).

On the other hand, group C has consistently greater prevalence of various neuropathology lesions. This trend was significant for group C individuals identified using tier 1 features for all amyloid‐associated and other, non‐cerebrovascular lesions (Figure 3, bolded diamond indicates significant mean *z*‐score). This included ADNC, Braak stage, density of diffuse and neuritic plaques, CAA, Lewy body disease, TDP‐43, and sclerosis of the hippocampus and/or MTL. Meanwhile, despite some cerebrovascular‐lesions having either lower or higher prevalence, the average *z*‐score of these lesions was not significantly different from the null due to the heterogeneous presentation. Detailed results for each group are presented in Figures S5–S7 in Supplemental Materials. In summary, groups A and C are two patient subgroups that were discovered using a purely data‐driven method and blinded to neuropathology by using only features from tier 1 or tiers 1–2. Despite this, groups A and C represent subpopulations that ultimately have either significantly lower or higher prevalence of specific neuropathologic lesions that are consistent across different disease domains.

### 3.4 Predicting neuropathology lesions using semi‐supervised machine learning

**FIGURE 4:**
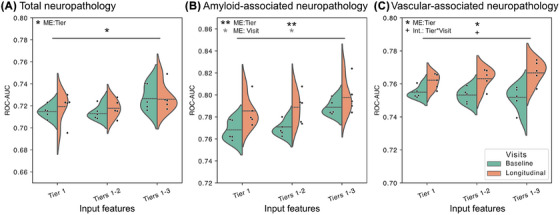
Predicting neuropathology load using differing tiers of feature and number of visits revealed high performance using lower burden data. A: For total neuropathology load, there was a main effect of tier. B: For predicting amyloid‐associated neuropathology load, main effects of tier and visit were both significant. C: For cerebrovascular neuropathology load, there was a main effect of tier and interaction effect of tier with visit. For all predictions, the main effect of tier only persists with a single clinical, indicating that neuropathology predictions using tier 1 with longitudinal visits were comparable to those using tiers 2 and 3 with a single visit. The significance of the interaction between feature tier and visits suggests that predictions of cerebrovascular neuropathology is most dependent on additional information across both data modality and time. *p < 0.05 and **p < 0.01 for main effects of tier and visit; +*p* < 0.05 for interaction effect between tier and visit. ME, main effect.

To further assess the diagnostic utility of low‐burden features, we developed prediction models using a semi‐supervised deep generative framework to leverage all the information available. Figure 4 presents our findings for predicting three key outcomes: total neuropathology load, amyloid‐associated neuropathology load, and cerebrovascular‐associated neuropathology load. We further broke down the performance for individual lesions in Figure S8 in Supplemental Materials. We trained the classifier using labeled and unlabeled data using different feature tiers either with a single visit or longitudinal data. We then used a two‐way repeated measures analysis of variance (ANOVA) to assess the impact of these two factors, feature tier and longitudinal data, on prediction performance reported as the ROC‐AUC. For predicting total load, we observed a significant main effect of tiers (F[2,8] = 10.67, *p* = 0.020), but no effect of including longitudinal information (*p* = 0.68). For amyloid‐associated load, we found significant main effects of both tiers (F[2,8] = 25.41, *p* = 0.0034) and longitudinal information (F[1,4] = 12.34, *p*‐value = 0.025). These trends were reflected in predictions of individual lesions, and the main effect of tier was most prominent across lesions with the exception of CAA, which did not have any significant main effects. In contrast, for predicting cerebrovascular‐associated load, there was no reliable effect of tiers (*p* = 0.74), but there was a reliable effect of including longitudinal information (F[1,4] = 9.482, *p* = 0.037) and an interaction (F[2,8] = 5.889, *p* = 0.032).

In general, adding more feature tiers improved prediction performance, but our *post‐hoc* analysis using an one‐way ANOVA revealed that this trend was only reliable when a single, baseline visit was available (one visit: total load, F[2,12] = 4.367, *p =* 0.038; longitudinal: total load, F[2,12] = 1.103, *p =* 0.36). This suggests that having tier 1 data at multiple clinical visits can functionally provide the same predictive abilities for overall neuropathology burden as having more data at one clinical visit. More specifically, when using tier 1 with longitudinal features, we obtained mean ROC‐AUC values of 0.719 for predicting total lesion load, 0.785 for predicting amyloid‐associated lesion load, and 0.762 for predicting cerebrovascular‐associated lesion load. In comparison, we obtained similar, respective values of 0.714, 0.769, and 0.753 when using tiers 1–2 without longitudinal features, and values of 0.728, 0.789, and 0.751 when using tiers 1–3 without longitudinal features. These results suggest that when participants have at least 3 clinical visits, tier 1 information can predict neuropathology load on par with (or better than) using tiers 2 and 3 information from a single visit. Since randomly guessing would yield a ROC‐AUC value of 0.5, our results were significantly better than chance and comparable to previous studies using higher burden data modalities.^14^

We also discovered that adding in longitudinal information did significantly improve performance for predicting amyloid‐associated load and cerebrovascular‐associated load (Figure 4B,C). For predictions that include tiers 3 features, adding in additional longitudinal information did not significantly alter performance for amyloid‐associated load. However, predictions of cerebrovascular‐associated load had the most significant improvement when both added feature tiers and longitudinal information were present. These findings indicate that cerebrovascular‐associated neuropathology requires the most intensive resources to accurately predict and would benefit from having both higher burden information and longitudinal data to properly assess.

To further understand the nature of the model's prediction errors, we analyzed additional prediction metrics (Table S3) and found that the model mostly excelled at minimizing false positive predictions with an overall high positive predictive value (PPV) or precision. On the other hand, the model struggled more to minimize false negative predictions. The precision and F1‐score of the model did not significantly change with the addition of higher burden features. These metrics were generally greater for amyloid‐associated and cerebrovascular‐associated lesions compared to total neuropathology load, suggesting that the model performed better when targeting specific domains of neuropathology rather than predicting overall pathology burden. The difference in some of the trends for the F1‐score and the ROC‐AUC values stems from how the two metrics handle the imbalance of class labels present in the dataset. Typically, the F1‐score yields a more “balanced” results across all class labels while the ROC‐AUC reflects the neuropathology load distributions in the dataset, which are slightly imbalanced. Here, we care equally about all neuropathology class labels (low, medium, and high load), so using the ROC‐AUC metric helps provide a more holistic evaluation of model performance.

### 3.5 Investigating important, low‐burden features for predicting neuropathology

To assess how low‐burden information impacts the prediction results from our semi‐supervised deep generative model, we investigated feature importance using leave‐one‐covariate‐out importance. If all tier 1 features are similarly important, then we would expect a flat distribution of feature importance values above chance. On the other hand, if only a small subset of features is important, then we would observe a set of features with high importance followed by a steep decline in feature importance values for the rest of the features that hover around chance. In this study, we observed a stepwise distribution of feature importance values for tier 1 features across all prediction tasks (Figure S9). A small group of features seemed to contribute the most meaningful information in each prediction task, but there were also secondary subsets of features that were moderately important. The top features tended to differ for each prediction task. Across all neuropathology prediction tasks, the GDS was consistently among the top 50 features, including asking if the participant dropped many of their activities/interests and the total GDS score (Table S4). Across most of the prediction tasks, demographics (e.g., level of independent, type of residence), behavioral surveys (including the GDS, FAS, NPI‐Q, and subjective report of memory decline), and medication/health history (including use of beta‐blockers, antipsychotics, and vision ability) were consistently among the top features. These findings suggest that neuropathology load may be linked to behavioral changes and comorbid medical conditions that should be further explored.

## 4 DISCUSSION

We investigated the relationship between clinical information, especially low‐burden features, and dementia neuropathology to arrive at four key findings. First, amyloid‐related neuropathology appeared to be dissociated from cerebrovascular‐related neuropathology as evidenced by both different load accumulation patterns (Figure S2) and distinctly clustered correlations (Figure 2A). Second, only a few key clinical features had significant associations with neuropathology, including neuropsychiatric and functional activities domains for tier 1 (Figure 2C‐D), Trails B for tier 2 (Figure 2E), and all tier 3 features with non‐cerebrovascular type lesions (Figure 2F). Third, we identified neuropathology‐enriched and neuropathology‐scarce subgroups of clinical relevance using low‐burden and generalizable clinical data (Figure 3). Fourth, we discovered that the inclusion of longitudinal information enabled tier 1 features on their own to perform on par with using more costly clinical information (Figure 4). Together, these findings demonstrate the utility of low‐burden data in dementia neuropathology screening and potential clinical trial enrichment.

Some of our findings highlight a major challenge in using low‐burden data to infer neuropathology as the simple correlations between tier 1 data and postmortem neuropathology lesions are weak. Time gaps between data collection and autopsy, brain resilience, and individual differences, among other factors, likely play important roles in affecting clinical features that may not map directly onto underlying neuropathology.^42^, ^43^ These challenges motivate methodologies that leverage longitudinal data to gain more informative signals and novel computational methods of inferring pathology status. Our work touches on both ideas to provide interpretable results for guiding potential clinical decision making. Contextualizing our findings in clinical practice, the prediction results from our model (PPV: 0.706‐0.994) generally exceeded the reported PPV metrics when comparing clinical diagnosis with neuropathology from the NACC dataset (0.46‐0.833).^28^ While we cannot directly compare results due to methodological differences, this suggests that our model performed at a clinically useful level, especially considering that all of our ROC‐AUC values were greater than 0.7.^44^

The representative clustering method identified a neuropathology‐enriched group and a neuropathology‐scarce group using slightly different input features. Tier 1 features helped identify a group of neuropathology‐enriched subjects and the addition of tier 2 features helped identify a group of neuropathology‐scarce subjects. This suggests that tier 1 features may be more informative at ruling in significant neuropathology burden while the addition of tier 2 features (i.e., neuropsychological testing) may be more useful for ruling out significant neuropathology burden. While we identified these significant clinical subgroups of interest, a considerable level of heterogeneity still exists in the study population. Cerebrovascular‐associated lesions tended to have the most variability across each group of subjects (Figure S6). Tier 1 features were not useful for inferring meaningful trends for presence of infarcts, microinfarcts, hemorrhages and microbleeds, and arteriolosclerosis. They were, however, useful for identifying significant trends in presence of atherosclerosis and white matter rarefaction, suggesting that these latter lesion types may have more widespread, behavioral effects corresponding to tier 1 information. Tier 2 features picked up on meaningful changes in cerebrovascular‐associated lesions except for hemorrhages and microbleeds, suggesting that additional data modalities, such as imaging, may be needed to accurately capture this neuropathology. In contrast, many trends were consistently observed and statistically significant across all other lesions. Importantly, the clusters and subsequent subject groups rely only upon a limited set of a priori selected lower burden features from participants without autopsy data to effectively identify enriched subgroups with greater prevalence of specific neuropathology lesions. These findings showcase the importance of learning from a large and more diverse patient sample to enable better contextualization of findings.

This work also demonstrated the utility of a semi‐supervised machine learning approach to improve the sample size, limited by brain donations, and reduce bias in the sample. Past work using semi‐supervised methods have been predominantly focused on multimodal imaging data for predicting clinical diagnosis,^45^, ^46^, ^47^ with little known about how this approach would benefit neuropathology prediction. Building upon other studies that have focused on predictions of neuropathology,^14^, ^15^ our work is the first to apply semi‐supervised learning to predict neuropathology lesions using low‐burden clinical data. We report reliably accurate ROC‐AUC values (0.714–0.798) for predictions of neuropathology load across multiple domains, including total, amyloid‐associated, and cerebrovascular‐associated neuropathology load. Future work should explore additional models and datasets that may improve generalizability of findings, including data from All of Us and Medicare Annual Wellness Visits.^48^, ^49^

We identified important features from the semi‐supervised machine learning model and summarized the top features for predicting total neuropathology load, amyloid‐associated lesions, and cerebrovascular‐associated lesions. Interestingly, the GDS (total score and certain questions), were consistently among the top features across most prediction tasks. This was followed by features from other behavioral surveys, such as the FAS and NPI‐Q, and other tier 1 features, including the use of a beta‐blocker or antipsychotic agent. The use of beta‐blockers, which are commonly prescribed to treat hypertension and essential tremors (among many other clinical indications), have been associated with greater vascular dementia risk over time.^50^ Furthermore, meta‐analyses suggest that beta‐blockers should not be first‐line agents for treating hypertension in individuals at risk of dementia and may have severe adverse effects in older adults.^51^, ^52^ Use of antipsychotics, which have been linked to increased risk of mortality, stroke, falls, and cognitive decline, has been studied by several groups and should be carefully considered in the older adult population.^53^, ^54^ Our work further corroborates the association of these medications with neuropathology. The use of these medications warrants further investigation for both researchers and clinicians working with older adults.

This study has several limitations. The data used are not representative of the general older adult population in the United States as they come from NACC and its associated network of ADRCs. The ADRCs are located at universities with advanced research programs, typically in heavily populated areas and they typically draw upon participants from the local community who wish to volunteer to aid research. Whether the biases associated with this sampling would affect our results is unclear and will need to be addressed in future work. Using postmortem neuropathology, instead of antemortem biomarkers of neuropathology, introduced additional noise in our understanding of disease onset in relation to the clinical features. These limitations were especially visible for certain neuropathology lesions, such as TDP‐43, which had a reduced sample size due to study design.^55^ We were also limited to certain clinical feature modalities in this study, which we hope future work will extend upon. In addition to our modeling approach, future analyses should explore transfer learning and multi‐task multi‐label classification approaches to capture more variability in low‐burden data.^56^ Future work should also explore additional data sets to validate our findings. By incorporating additional datasets in future studies, we can enhance the generalizability of our findings and assess whether specific models offer better out‐of‐distribution performance. Additional datasets, such as the National Centralized Repository for Alzheimer's Disease and Related Dementias and the Alzheimer's Disease Neuroimaging Initiative, can be included as more participant data are collected in the coming years.

As plasma biomarkers become widely available, our work can benefit from multimodal integration of plasma biomarkers with low‐burden clinical data. Given the difficulties in using existing clinical data to predict neuropathology, plasma biomarkers would be uniquely suited to synergize with this framework to distinguish different neuropathology types more sensitively. Rather than needing to identify a singular, best biomarker, our work demonstrates the effectiveness of integrating different domains of low‐burden data for disease prediction to improve healthcare access and outcomes. As precision health becomes more accessible, additional modalities of low‐burden data will become increasingly valuable. Beyond plasma biomarkers, these include lifestyle factors, physical activity levels, cognitive biomarkers, and digital biomarkers.^57^, ^58^, ^59^ Future work should seek to integrate various sources of low‐burden features to improve precision health in predicting neuropathology. While low‐burden data can be highly useful, our results emphasized the need for longitudinal data collection. With at least three clinical visits, the prediction performance using tier 1 features was equivalent to that of using tiers 2 or 3 features. This demonstrates the need to integrate multiple time points of clinical information in future work, which may provide additional challenges as different modalities of data may have different collection time points and intervals. Additionally, our prediction results highlight an ongoing challenge in the field to accurately capture cerebrovascular‐associated neuropathology using existing modalities of data. While low‐burden features can predict amyloid‐associated neuropathology relatively well, we lack low‐burden information that can yield similarly accurate predictions of cerebrovascular neuropathology despite having features such as blood pressure and cerebrovascular history. Our findings highlight the need for the development of more sensitive, accessible biomarkers to capture specific measures of cerebrovascular‐associated dementia neuropathology.

Our framework for identifying enriched subgroups for different neuropathology lesions using low‐burden data may present opportunities for screening in clinical trials, not limited to pathologies that have existing positron emission tomography (PET) tracers. This would reduce barriers to recruitment and enrollment, ultimately improving representation in clinical trials. Our work serves as a first step toward this goal, and future work should focus on engineering data‐driven models that can generalize to broader populations while improving upon specificity of the neuropathology lesions of interest.

## CONFLICT OF INTEREST STATEMENT

The authors declare no conflicts of interest. Author disclosures are available in the supporting information.

## CONSENT STATEMENT

All human subject data used in this study were obtained from NACC and recorded such that subjects cannot be identified. Due to this, consent was not necessary for the use of human subject data in this study.

## Supporting information



Supporting Information

Supporting Information
