# Supplementary material for: Identifying dementia neuropathology using low‐burden clinical data
Source: Alzheimers Dement. 2025 Aug 4;21(8):e70539. doi: 10.1002/alz.70539 (PMC12322317; doi:10.1002/alz.70539)
Supplement: Supplementary file 1 — Supporting Information [file ALZ-21-e70539-s001.docx]

**Supplemental Materials**

**Methods**

The sample for this study includes information from 46 Alzheimer’s Disease Research Centers (ADRCs) collected from September 2005 to March 2023. We excluded participants with more than 50% missing information after feature processing. We did not exclude any participants based upon clinical status. In generating the T- Data Set, and to better assess longitudinal clinical data, as most clinical visits occur annually, we created year-long time windows measured from the date of death for each participant to identify approximately equally spaced clinical visits up to 7 years before autopsy (Figure S1). This yielded longitudinal UDS visits for participants aged around 60 years or older prior to autopsy with similar sample sizes for each visit year. To ensure a minimum number of longitudinal visits for meaningful trends, we excluded subjects with fewer than three UDS visits.

We explored various other datasets that could enhance our sample and provide additional feature modalities, including the National Centralized Repository for Alzheimer’s Disease and Related Dementias (NCRAD) and the Alzheimer’s Disease Neuroimaging Initiative (ADNI), among many datasets through the Global Alzheimer's Association Interactive Network (GAAIN) online portal. At the time of analysis, there were not enough participants in NCRAD to justify inclusion of fluid biomarkers, especially blood biomarkers, and not enough neuropathology data available in ADNI to include as an additional, independent cohort. In future work, we would like to focus on adding more datasets and feature modalities, including exploring the connections between genetic data, clinical information, and neuropathology through datasets such as the Mount Sinai/JJ Peters VA Medical Center Brain Bank (MSBB–Mount Sinai NIH Neurobiobank) cohort.^1^

Because the Neuropathology Data Set does not have an overall measure of AD-related neuropathologic change (ADNC), we defined an additional lesion variable for ADNC as the presence of either moderate to high level of Braak staging with evidence of moderate to high density of neuritic plaques or evidence of moderate to high density of diffuse cortical plaques, following definitions from past work.^2,3^ Data for some of these lesions were collected on an ordinal scale of varying disease severity, and to streamline the subsequent analyses, we decided to binarize the lesions as being either absent (none to low level) or present (moderate to high level) in the participant following the same procedure as outlined previously.^3^ Doing so allowed us to tabulate the neuropathology burden, or cumulative neuropathology load, for each participant as a sum of lesions present. These lesions are described in Table S1.

For total neuropathology load, class thresholds were identified by finding the 20^th^ and 80^th^ percentiles, which corresponded to 0-4 (none/low), 5-7 (medium), and 8-14 (high). Other cutoffs were explored, but while alternative thresholds yielded more equal class samples, they also resulted in unreasonable cutoff points that defined a class as a single load value (such as the mode, 7). We used domain knowledge to guide these decisions, and for amyloid-associated neuropathology load, we defined the thresholds as 0-1 (none/low), 2-3 (medium), and 4-5 (high). Because we included ADNC as an amyloid-associated lesion, these load cutoffs ensure that subjects with none/low load do not have ADNC present, while subjects with medium loads may have ADNC, and subjects with high load always have ADNC. For cerebrovascular-associated neuropathology load, there were many participants with no positive lesions (as defined by none/low values before binarization). Thus, we defined the cutoffs to be 0 (none/low), 1-2 (medium) and 3-5 (high) to capture the variation and maintain relatively balanced class sizes. These cutoffs are illustrated in Figure 1E.

For data processing, we first removed features that have excessive missingness greater than 30% to 40% of subjects, depending on the data modality, to ensure that we can align the features in each tier closely with our past work.^4^ To this end, we used the Crosswalk Study to convert newer neuropsychological testing scores to a set of common testing features to reduce missingness.^5^ All Uniform Data Set (UDS) features used are listed in Table S2 in Supplemental Materials. Next, we removed subjects with more than 50% missing features. For each type of feature, including categorical, ordinal, and numerical, we encoded the feature values appropriately and performed single-value imputation. In detail, this means that we performed one-hot encoding for categorical features and ordinal encoding for ordinal features to preserve feature ordering. To ensure that demographic effects were not driving differences in neuropsychological testing features, we regressed out age, sex, and years of education from these numerical features.

We stratified UDS features into tiers based upon burden, accessibility, and cost. Tier 1 includes information from medical history, patient interviews, and routine screening questions that we proxied using the corresponding features from the UDS (e.g., using the Geriatric Depression Scale in the UDS to capture the Patient Health Questionnaire-2 commonly used in practice). We included the Mini-Mental State Exam (MMSE) as a Tier 1 feature due to its abundant use in primary care settings.^6^ Tier 2 features are medium-burden, and this corresponded to features from a full battery of neuropsychological testing. Due to the change in the neuropsychological testing battery between different versions of the UDS, we selected a set of overlapping features from the Crosswalk Study that enabled us to convert scores on the newer tests into scores on the older tests, which were more abundant.^5^

To generate longitudinally derived features for the T- Data Set, we processed each type of clinical information separately. For categorical features, we included the mode and change rate (number of times the feature value changed over the number of visits available). For ordinal features, we included the mean, change rate, max change over time (maximum difference in feature values over the number of visits available), and area under the curve of the longitudinal data points. For numeric features, we included the mean, variance, max change, slope, and area under the curve. For all subjects, we further included the number of visits available as an additional feature.

In our clustering analysis, we explored a wide range of methodologies. As the number of features increases, dimensionality reduction is an important first step to address the curse of dimensionality for finding subgroups in a high-dimensional space. We compared various dimensionality reduction methods prior to applying clustering, including feature selection methods, including Least Absolute Shrinkage and Selection Operator (LASSO)^7^ and *a priori* features selected from our past work.^4^ We first selected *a priori* features from the top tier 1 features, included age, sex, and education, then included additional tier 2 neuropsychological testing features for analyses using tiers 1-2 features (Table S3).^4^ Additionally, we examined dimensionality reduction techniques such as principal component analysis^8^ and autoencoder.^9,10^ When we compared across dimensionality reduction methods, we found that while clustering quality did not differ much, using *a priori* features followed by an autoencoder improved clustering-based classification of clinical diagnoses, which served as a benchmark for finding clinically distinct groups.^4^ Intuitively, this approach allowed the supervised feature selection step to reduce noise prior to feature extraction.^11^ Consequently, the clusters we obtained spanned varying levels of clinical severity and cognitive impairment. We tuned the autoencoder using Optuna, a hyperparameter tuning framework, to find the ideal network architecture and training setup.^12^

We compared two clustering methods, a Gaussian mixture model (GMM) and a variational Bayesian estimation of a GMM using a Dirichlet process for priors to infer the number of clusters from the data, both implemented in scikit-learn using Python.^13^ We conducted hyperparameter tuning for each method, especially for the maximum number of clusters, by calculating the silhouette score, a measure of clustering quality,^14^ using a variety of different distance metrics, including Cosine similarity, Manhattan distance, Minkowski distance, Mahalanobis distance, Euclidean distance, and average distance.^15^ We then selected the hyperparameters that yielded the highest silhouette score using any distance metric, as each metric captures slightly different aspects of cluster quality. We discovered that having three clusters was the most optimal setup for both clustering methods across a wide range of hyperparameters. Because clustering quality did not significantly change between GMM and Bayesian GMM, we selected GMM for its ease of computation.

For each prediction pipeline, we conducted 5-fold cross validation. After feature processing, we rebalanced the classes by oversampling the minority class either using a random oversampling method for supervised classifiers (implemented in Python using imbalanced-learn)^16^ or weighing minority class instances more heavily for the semi-supervised classifier (implemented using Pyro).^17^ We compared this approach to using Synthetic Minority Oversampling Technique (SMOTE),^18^ a popular machine learning technique to account for class imbalances, and did not observe a significant difference in results. Next, we performed dimensionality reduction and opted for univariate feature selection methods, as the classifiers themselves also perform some levels of dimensionality reduction. We compared feature selection methods using analysis of variance (ANOVA) and mutual information. We found that ANOVA-based feature selection performed the best in hyperparameter tuning trials implemented in Optuna. We also compared the performance of popular supervised methods, including logistic regression,^19^ random forest,^20^ and gradient boosting (implemented using XGBoost)^21^, against our implementation of a classic semi-supervised deep generative model using a variational autoencoder (VAE) and deep neural network implemented using Pyro.^22^ All other models vastly outperformed the benchmark logistic regression model, with random forest, gradient boosting, and semi-supervised model performing similarly to each other. We proceeded to report two-way ANOVA results from the semi-supervised model due to its greater generalizability gained from training using participant data broadly, not limited to the NP Data Set. The *p*-value results were adjusted using the Greenhouse-Geisser correction to reduce type 1 error for lack of sphericity.^23^

**Tables**

**Table S1. Description of T- Data Set.**

| Features | | Initial visit | Final visit |
| --- | --- | --- | --- |
| Age at death, year (SD) | | 83.9 (9.01) | |
| Sex (% female) | | 46.9 | |
| Age, year (SD) | | 78.6 (8.88) | 82.7 (9.06) |
| Cognitive status (%) | Cognitively unimpaired | 26.3 | 14.6 |
|  | Subjective cognitive decline | 2.44 | 1.47 |
|  | Mild cognitive impairment | 17.7 | 9.72 |
|  | Dementia | 53.6 | 74.2 |
| CDR Global Score (%) | 0 | 26.2 | 14.1 |
|  | 0.5 | 34.1 | 15.9 |
|  | 1 | 24.9 | 14.5 |
|  | 2 | 9.49 | 20.6 |
|  | 3 | 5.28 | 34.9 |
| ApoE genotype (%) | e3/e3 | 45.9 | |
|  | e3/e4 | 29.2 | |
|  | e3/e2 | 7.95 | |
|  | e4/e4 | 7.32 | |
|  | e4/e2 | 2.67 | |
|  | e2/e2 | 0.398 | |
|  | Unknown | 6.48 | |
| Alzheimer’s disease neuropathologic change (%) | Negative | 11.9 | |
|  | Positive | 86.4 | |
|  | Unknown | 1.74 | |
| Cerebral amyloid angiopathy (%) | Negative | 67.8 | |
|  | Positive | 30.6 | |
|  | Unknown | 1.57 | |
| Infarcts (%) | Negative | 80.7 | |
|  | Positive | 18.7 | |
|  | Unknown | 0.623 | |
| Microinfarcts (%) | Negative | 77.3 | |
|  | Positive | 22.3 | |
|  | Unknown | 0.399 | |
| Lewy body disease (%) | Negative | 64.7 | |
|  | Positive | 34.7 | |
|  | Unknown | 0.623 | |
| Hippocampal and/or medial temporal lobe sclerosis (%) | Negative | 83.0 | |
|  | Positive | 13.4 | |
|  | Unknown | 3.54 | |

**Table S2. Clinical features derived from the Uniform Data Set.**

| ABUSOTHR | CVANGIO | GAMES | MEMUNITS | NACCHTNC | RESIDENC |
| --- | --- | --- | --- | --- | --- |
| AFRAID | CVBYPASS | HALLSEV | MOTSEV | NACCLIPL | SATIS |
| AGITSEV | CVCHF | HAPPY | NACCAAAS | NACCLIVS | SEIZURES |
| ALCOHOL | CVHATT | HEARAID | NACCAANX | NACCMMSE | SEX |
| ANIMALS | CVOTHR | HEARING | NACCAC | NACCMOM | SHOPPING |
| ANXSEV | CVPACE | HEARWAID | NACCACEI | NACCNIHR | SMOKYRS |
| ANYMEDS | DECSUB | HEIGHT | NACCADEP | NACCNREX | SPIRITS |
| APASEV | DELSEV | HELPLESS | NACCAGE | NACCNSD | STAYHOME |
| APOERISK | DEP2YRS | HISPANIC | NACCAHTN | NACCPDMD | STOVE |
| APPSEV | DEPDSEV | HOMEHOBB | NACCAMD | NACCSTYR | TAXES |
| B12DEF | DEPOTHR | HOPELESS | NACCANGI | NACCTBI | THYROID |
| BETTER | DIABETES | HRATE | NACCAPSY | NACCTIYR | TOBAC100 |
| BILLS | DIGIB | HYPERCHO | NACCBETA | NACCVASD | TOBAC30 |
| BORED | DIGIBLEN | HYPERTEN | NACCCCBS | NCOTHR | TRAILA |
| BOSTON | DIGIF | INCONTF | NACCDAD | NITESEV | TRAILB |
| BPDIAS | DIGIFLEN | INCONTU | NACCDBMD | NOGDS | TRAVEL |
| BPSYS | DISNSEV | INDEPEND | NACCDIUR | ORIENT | VEG |
| CBSTROKE | DROPACT | IRRSEV | NACCEMD | PACKSPER | VISCORR |
| CBTIA | EDUC | JUDGMENT | NACCEPMD | PAYATTN | VISION |
| CDRGLOB | ELATSEV | MARISTAT | NACCFADM | PERSCARE | VISWCORR |
| CDRSUM | EMPTY | MEALPREP | NACCFAM | PRIMLANG | WEIGHT |
| COMMUN | ENERGY | MEMORY | NACCFFTD | PSYCDIS | WONDRFUL |
| CVAFIB | EVENTS | MEMPROB | NACCGDS | REMDATES | WRTHLESS |

**Table S3. Prediction performance metrics for neuropathology load.**

| Prediction task | Tier | Visits | PPV (SD) | F1-score (SD) | ROC-AUC (SD) |
| --- | --- | --- | --- | --- | --- |
| Total load | 1 | Baseline | **0.754 (0.0382)** | 0.607 (0.00674) | 0.715 (0.00581) |
|  |  | Longitudinal | 0.706 (0.0872) | 0.592 (0.0364) | 0.719 (0.0135) |
|  | 2 | Baseline | 0.721 (0.0600) | 0.595 (0.0163) | 0.714 (0.00629) |
|  |  | Longitudinal | 0.715 (0.0639) | 0.590 (0.0215) | 0.718 (0.00807) |
|  | 3 | Baseline | 0.721 (0.0451) | 0.610 (0.0132) | **0.728 (0.0117)** |
|  |  | Longitudinal | 0.722 (0.0549) | **0.611 (0.0232)** | **0.728 (0.0125)** |
| Amyloid-associated load | 1 | Baseline | **0.892 (0.0830)** | **0.696 (0.0281)** | 0.767 (0.00909) |
|  |  | Longitudinal | 0.744 (0.0265) | 0.658 (0.0142) | 0.785 (0.0137) |
|  | 2 | Baseline | 0.852 (0.0757) | 0.683 (0.0300) | 0.769 (0.00671) |
|  |  | Longitudinal | 0.726 (0.0552) | 0.650 (0.0238) | 0.789 (0.0141) |
|  | 3 | Baseline | 0.770 (0.0659) | 0.670 (0.0259) | 0.790 (0.00695) |
|  |  | Longitudinal | 0.707 (0.0200) | 0.649 (0.0163) | **0.798 (0.0154)** |
| CV-associated load | 1 | Baseline | **0.994 (0.0134)** | 0.757 (0.00664) | 0.755 (0.00352) |
|  |  | Longitudinal | 0.988 (0.0217) | **0.762 (0.0118)** | 0.762 (0.00455) |
|  | 2 | Baseline | 0.981 (0.0260) | 0.751 (0.0126) | 0.753 (0.00449) |
|  |  | Longitudinal | 0.973 (0.0338) | 0.756 (0.0160) | 0.763 (0.00600) |
|  | 3 | Baseline | 0.980 (0.0402) | 0.750 (0.0197) | 0.751 (0.00731) |
|  |  | Longitudinal | 0.987 (0.0235) | **0.762 (0.0114)** | **0.767 (0.00691)** |

**Table S4. Top tier 1 features across neuropathology load predictions.**

| Included as top feature | Domain | Features |
| --- | --- | --- |
| In 6 prediction tasks | Behavioral surveys (e.g., GDS) | Dropped any activities (DROPACT), total GDS score (NACCGDS) |
| In 5 prediction tasks | Demographics (e.g., living situation) | Level of independence (INDEPEND) |
|  | Behavioral surveys (e.g., GDS, FAQ) | Lack of GDS score (NOGDS), remembering dates (REMDATES), subjective report of memory decline (DECSUB) |
|  | Patient history (e.g., medications) | Use of beta-blocker (NACCBETA) |
| In 4 prediction tasks | Demographics (e.g., living situation) | Type of residence (RESIDENC) |
|  | Behavioral surveys (e.g., GDS, FAQ, NPI-Q) | AFRAID, WONDRFUL, TAXES, PAYATTN, ANXSEV, AGITSEV |
|  | Patient history (e.g., medications) | Vision (VISWCORR), use of antipsychotic agent (NACCAPSY) |

**Figures**

**D
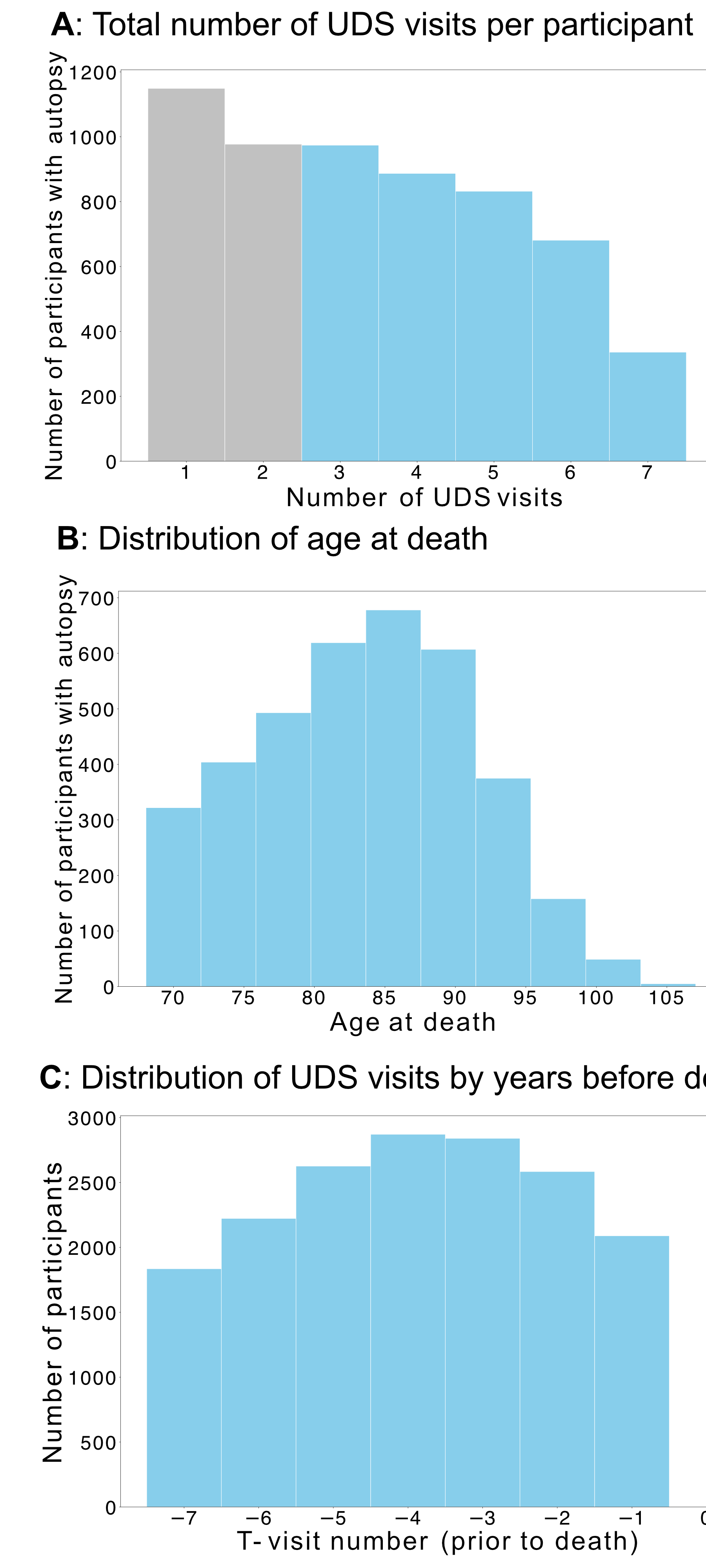
**

**Figure S1: Sample characteristics of T- Data Set. A:** The total number of UDS visits across subjects in our datasets, with grey bars indicating subjects with fewer than 3 visits (excluded from analysis). **B:** The distribution of the age at death for all subjects, peaking around 85 years. **C:** **D:** The distribution of UDS visits by year before autopsy, with most visits occurring around 3-5 years before death.

**
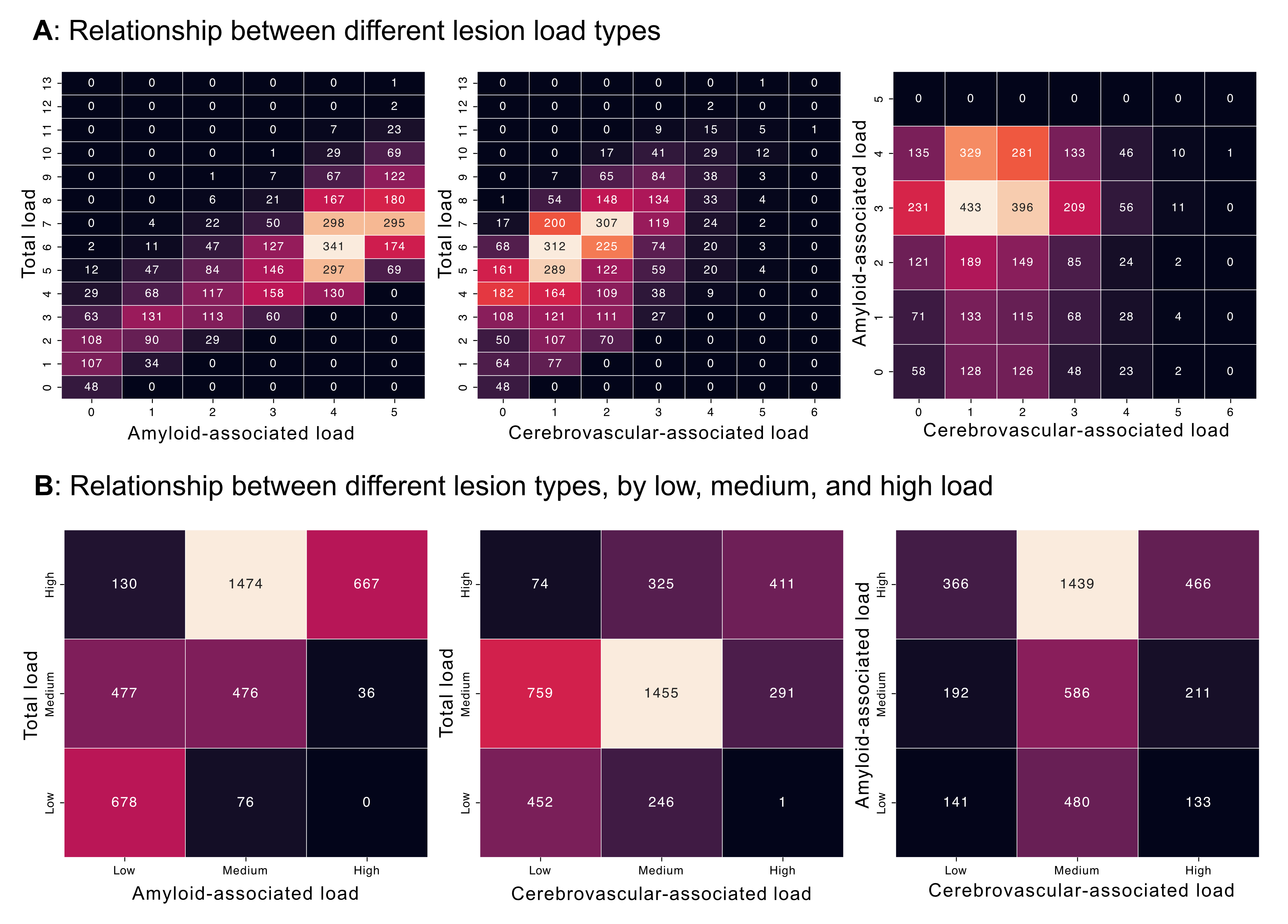
**

**Figure S2: Defining neuropathology burden as different load types.** To capture the burden of mixed neuropathologies, we defined three types of pathology load as the sum of pathologies in that domain and stratified subjects into low, mid, and high load classes for each load type. Amyloid-associated lesions include ADNC, BRAAK, NEUR, DIFF, CAA; cerebrovascular-associated lesions include INF, MICR, HEM, ARTE, AVAS, WMR. **A:** Visualizing the overlap of each load type demonstrated differences in how amyloid- and cerebrovascular-associated lesions accumulated with respect to overall neuropathology load. **B:** Similar trends were preserved when each load type was binned by low, mid, and high load classes.

**
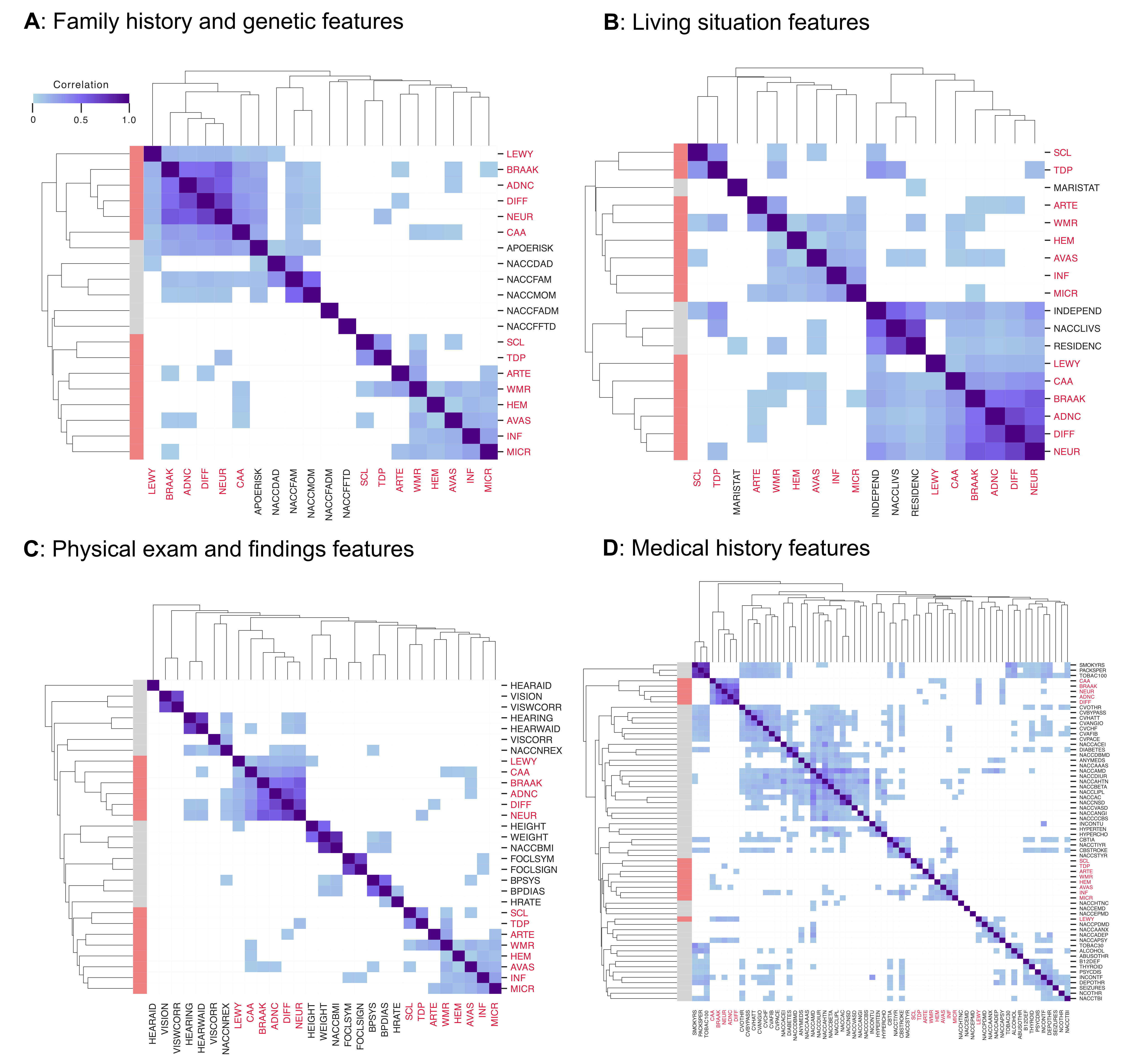
**

**Figure S3: Correlations between additional tier 1 feature modalities and neuropathology lesions. A:** Family history and genetic data revealed some significant associations with amyloid-associated lesions and Lewy body disease. **B:** Living situation features were all of the amyloid-associated lesions. **C:** A few physical exam features were associated with neuropathology, including focal neurological exam signs and systolic blood pressure with cerebrovascular-associated lesions. **D:** Some medical history features, including medications and history of cerebrovascular events, were significantly correlated with neuropathology. Pearson correlation coefficients with *p*-values < 0.05 after Bonferroni correction were plotted.

**
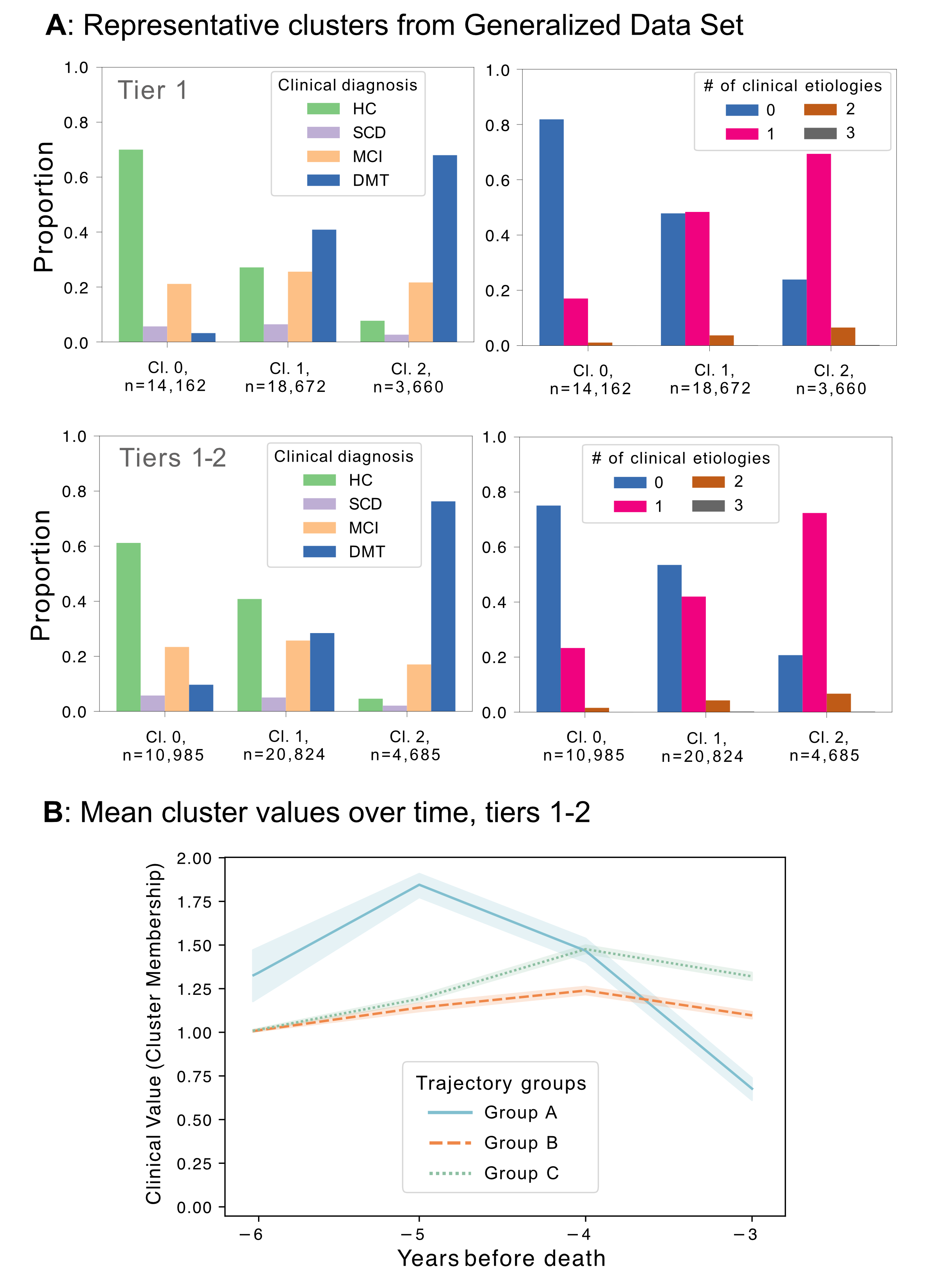
**

**Figure S4: Generalized Data Set clusters yielded three distinct subgroups of T- Data Set subjects over time. A:** Using subjects without autopsy from the Generalized Data Set, three representative clusters with varying levels of clinical impairment were created. Each cluster also showed differing complexity of disease etiologies diagnosed clinically. Similar clusters were found using either tier 1 only or tiers 1-2 features. **B:** Subjects with autopsy from the T- Data Set were assigned to the existing clusters at each time point available, generating three distinct trajectory subgroups. Each subgroup had differing levels of progressing to greater clinical impairment over time, excluding the immediate two years before death due to sample bias.

**
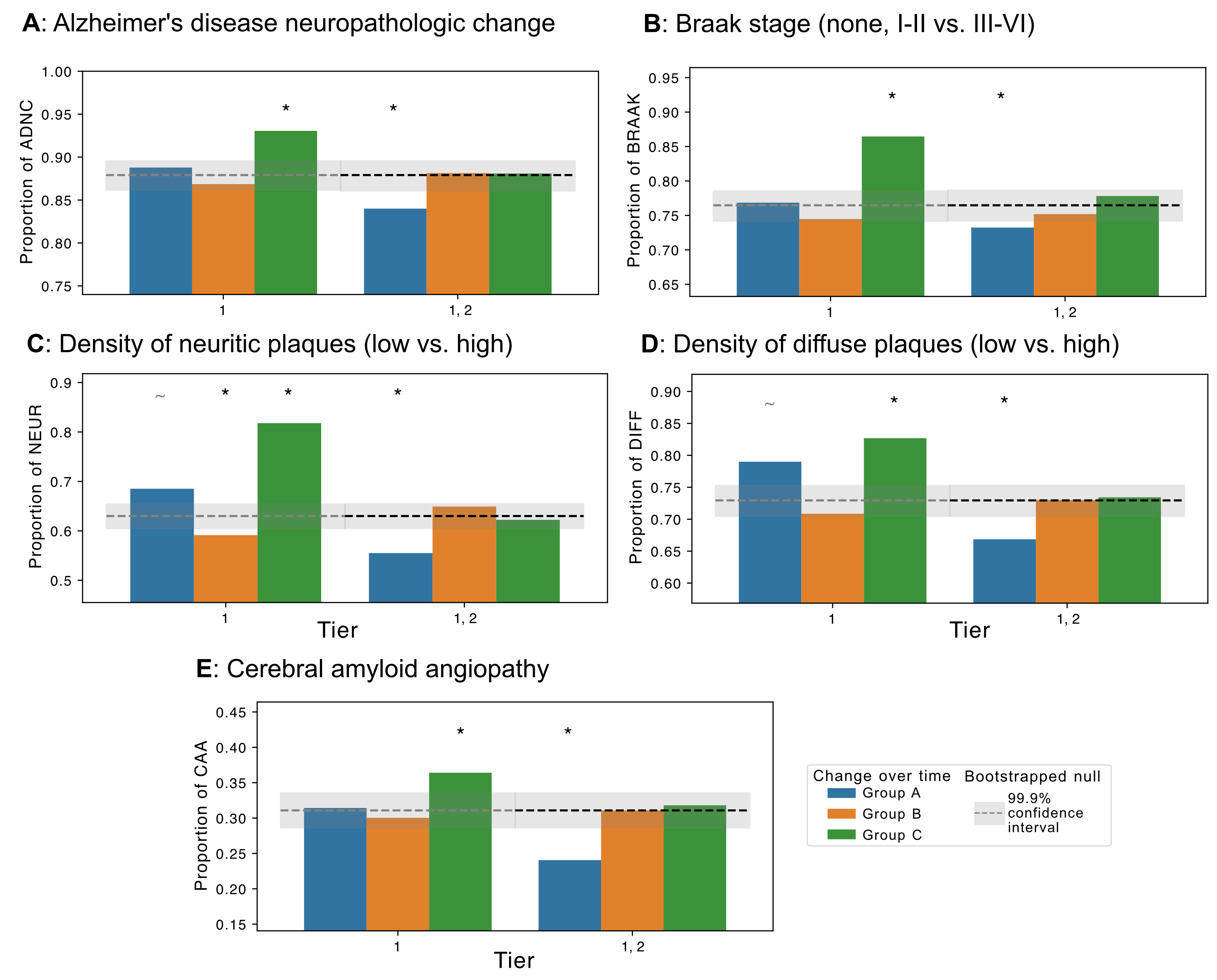
**

**Figures S5: Clustering subgroups revealed significantly different amyloid-associated neuropathology prevalence.** Using only tier 1 features, group C had significantly greater prevalence of all the amyloid-associated lesions, including Alzheimer’s disease neuropathologic change (ADNC), Braak stage, density of neuritic plaques, density of diffuse plaques, and cerebral amyloid angiopathy. Using tiers 1-2 features, group A had significantly lower prevalence of all the lesions above. * denotes values outside of bootstrapped 99.9% confidence interval, and ~ denotes subgroups with a sample size of less than 200.


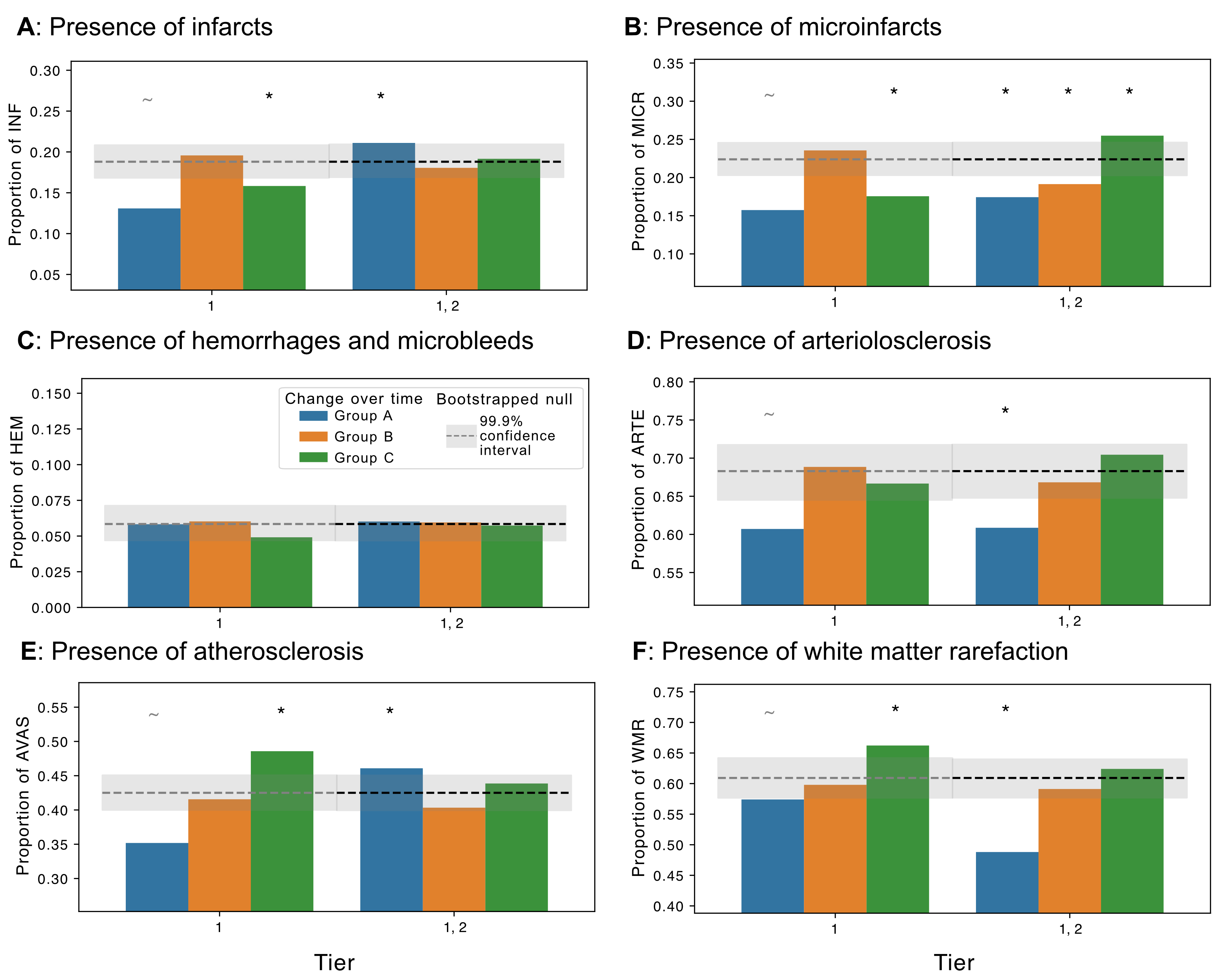


**Figures S6: Some clustering subgroups revealed significantly different cerebrovascular-associated neuropathology prevalence.** With only tier 1 features, group C had significantly greater prevalence of atherosclerosis and white matter rarefaction lesions. With tiers 1-2 features, group A had significantly lower prevalence of microinfarcts, arteriolosclerosis and white matter rarefaction, while group C had significantly greater prevalence of microinfarcts. Other neuropathology lesions, such as hemorrhages and microbleeds, did not have any significant trends. Overall, cerebrovascular-associated lesions had much more noise and less consistent trends with regards to subgroups having greater or lower prevalence of pathology. * denotes values outside of bootstrapped 99.9% confidence interval, and ~ denotes subgroups with a sample size of less than 200.


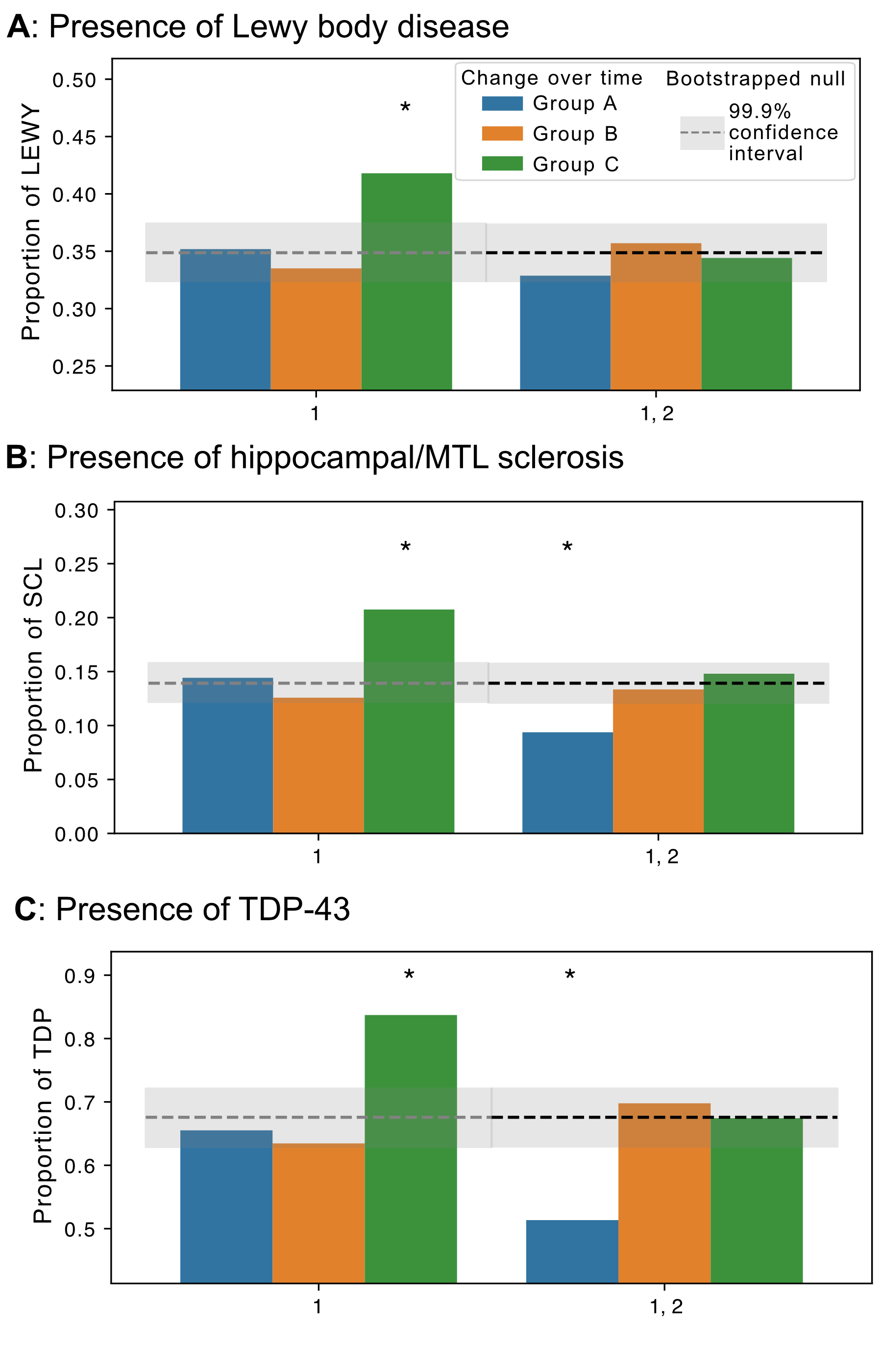


**Figures S7: Clustering subgroups consistently revealed differing neuropathology prevalence for Lewy body disease, hippocampal sclerosis, and TDP-43.** Using only tier 1 features, group C had significantly greater prevalence of all three lesions. Using tiers 1-2 features, group A had significantly lower prevalence of hippocampal/MTL sclerosis and TDP-43. These trends were quite consistent with that of the amyloid-associated neuropathology lesions and many of the cerebrovascular-associated lesions. * denotes values outside of bootstrapped 99.9% confidence interval, and ~ denotes subgroups with a sample size of less than 200.


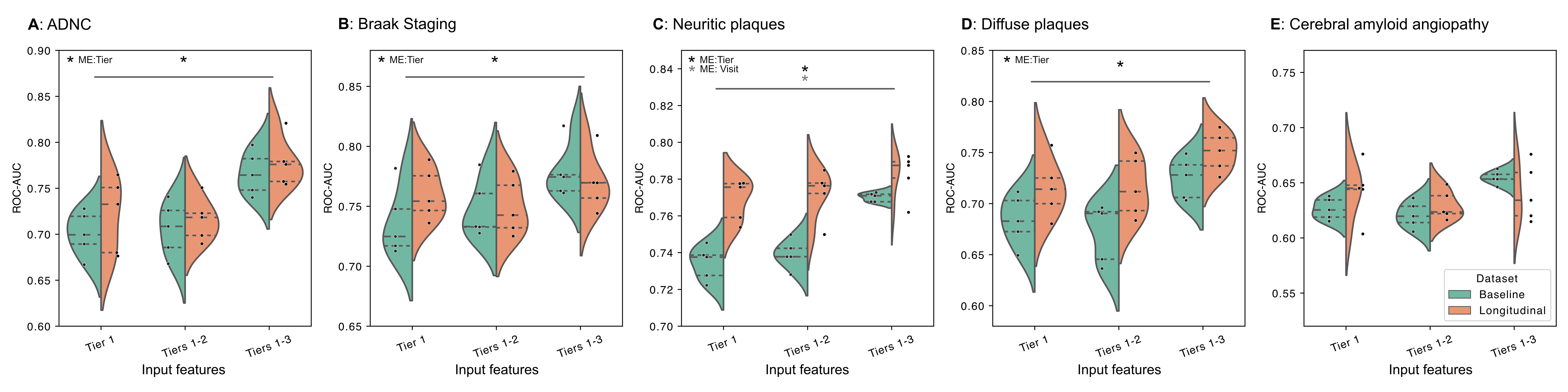


**Figure S8: Prediction results for amyloid-associated lesions using differing tiers of input features with and without longitudinal data.** Each amyloid-associated neuropathological lesion is presented as a binary prediction task using the same semi-supervised learning framework and pipeline. We found that almost all prediction outcomes were in a similar range of ROC-AUC values, with predictions of Alzheimer’s disease neuropathologic change (ADNC), Braak staging, and diffuse amyloid plaques performing the highest, and cerebral amyloid angiopathy (CAA) performing the lowest. Additional features from tiers 2 and 3 significantly boosted performance and adding in longitudinal information significantly enhanced predictions of neuritic amyloid plaques. **A:** For predictions of ADNC, we found a significant main effect of tier (F[2,8] = 69.37, *p* = 0.00035). **B:** For predictions of Braak staging, we reported a significant main effect of tier (F[2,8] = 54.12, *p* = 0.00041). **C:** For predictions of neuritic plaques, we found significant main effects of including longitudinal information (F[1,4] = 32.35, *p* = 0. 0047) and tier (F[2,8] = 18.85, *p* = 0.0035). **D:** For predictions of diffuse plaques, we found a significant main effect of tier (F[2,8] = 42.07, *p* = 0.00020). **E:** For predictions of CAA, we did not found a significant main effect.

**
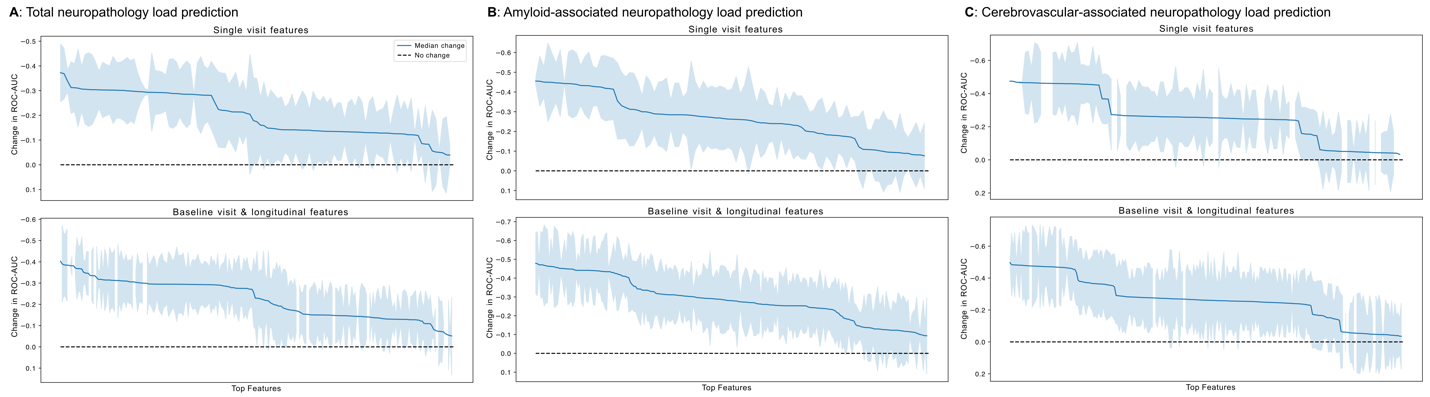
**

**Figure S9: Feature importance trends for tier 1 features across all prediction tasks.** Across all prediction tasks, a small group of features appear to contribute the most meaningful information, followed by secondary subsets of features that are moderately important. These trends reflect a stepwise change in feature importance across tier 1 features, suggesting that while some features contribute more meaningful information, having the context of additional features present is necessary for prediction performance.

**References**

1. Wang M, Beckmann ND, Roussos P, et al. The Mount Sinai cohort of large-scale genomic, transcriptomic and proteomic data in Alzheimer’s disease. *Sci Data*. 2018;5(1):180185. doi:10.1038/sdata.2018.185

2. Hyman BT, Phelps CH, Beach TG, et al. National Institute on Aging–Alzheimer’s Association guidelines for the neuropathologic assessment of Alzheimer’s disease. *Alzheimer’s &amp; Dementia*. 2012;8(1):1-13. doi:10.1016/j.jalz.2011.10.007

3. Phongpreecha T, Cholerton B, Bukhari S, et al. Prediction of neuropathologic lesions from clinical data. *Alzheimer’s & Dementia*. 2023;19(7):3005-3018. doi:10.1002/alz.12921

4. Ren Y, Shahbaba B, Stark CEL. Improving clinical efficiency in screening for cognitive impairment due to Alzheimer’s. *Alzheimers Dement Diagn Assess Dis Monit*. 2023;15(4):e12494. doi:10.1002/dad2.12494

5. Monsell SE, Dodge HH, Zhou XH, et al. Results From the NACC Uniform Data Set Neuropsychological Battery Crosswalk Study. *Alzheimer Disease & Associated Disorders*. 2016;30(2):134-139. doi:10.1097/WAD.0000000000000111

6. Alzheimer’s Association. 2019 Alzheimer’s disease facts and figures. Special report: Alzheimer’s detection in the primary care setting. *Alzheimers Dement*. 2019;15(3):321-387. doi:10.1016/j.jalz.2019.01.010

7. Tibshirani R. Regression Shrinkage and Selection via the Lasso. *JSTOR*. 1996;58(1):267-288.

8. Hotelling H. Analysis of a complex of statistical variables into principal components. *Journal of Educational Psychology*. 1933;24(6):417-441. doi:10.1037/h0071325

9. Rumelhart, D. E, Hinton, G. E., Williams, R. J. Learning internal representations by error propagation. In: *Parallel Distributed Processing: Explorations in the Microstructure of Cognition, Vol. 1: Foundations*. Vol 1. MIT Press; 1986:318-362.

10. Kumar V, Nandi GC, Kala R. Static hand gesture recognition using stacked Denoising Sparse Autoencoders. In: *2014 Seventh International Conference on Contemporary Computing (IC3)*. IEEE; 2014:99-104. doi:10.1109/IC3.2014.6897155

11. Trinh M, Shahbaba R, Stark CE, Ren Y. Alzheimer’s Disease Detection Using Data Fusion with a Deep Supervised Encoder. *Front Dement*. 2024;3. doi:10.3389/frdem.2024.1332928

12. Akiba T, Sano S, Yanase T, Ohta T, Koyama M. Optuna: A Next-generation Hyperparameter Optimization Framework. In: *Proceedings of the 25th ACM SIGKDD International Conference on Knowledge Discovery & Data Mining*. ACM; 2019:2623-2631. doi:10.1145/3292500.3330701

13. Pedregosa F, Varoquaux G, Gramfort A, et al. Scikit-learn: Machine Learning in Python. *Journal of Machine Learning Research*. 2011;12(85):2825-2830.

14. Rousseeuw PJ. Silhouettes: A graphical aid to the interpretation and validation of cluster analysis. *Journal of Computational and Applied Mathematics*. 1987;20:53-65. doi:10.1016/0377-0427(87)90125-7

15. Shirkhorshidi AS, Aghabozorgi S, Wah TY. A Comparison Study on Similarity and Dissimilarity Measures in Clustering Continuous Data. Dalby AR, ed. *PLoS ONE*. 2015;10(12):e0144059. doi:10.1371/journal.pone.0144059

16. Lemaitre G, Nogueira F, Aridas CK. Imbalanced-learn: A Python Toolbox to Tackle the Curse of Imbalanced Datasets in Machine Learning. *Journal of Machine Learning Research*. 2016;18(17):1-5.

17. Bingham E, Chen JP, Jankowiak M, et al. Pyro: Deep Universal Probabilistic Programming. Published online 2018. doi:10.48550/ARXIV.1810.09538

18. Chawla NV, Bowyer KW, Hall LO, Kegelmeyer WP. SMOTE: Synthetic Minority Over-sampling Technique. *jair*. 2002;16:321-357. doi:10.1613/jair.953

19. D.R. Cox. The Regression Analysis of Binary Sequences. *Journal of the Royal Statistical Society*. 1958;20:215-242.

20. Breiman L. Random Forests. *Machine Learning*. 2001;45(1):5-32. doi:10.1023/A:1010933404324

21. Chen T, Guestrin C. XGBoost: A Scalable Tree Boosting System. In: *Proceedings of the 22nd ACM SIGKDD International Conference on Knowledge Discovery and Data Mining*. ; 2016:785-794. doi:10.1145/2939672.2939785

22. Kingma DP, Rezende DJ, Mohamed S, Welling M. Semi-Supervised Learning with Deep Generative Models. Published online 2014. doi:10.48550/ARXIV.1406.5298

23. Abdi H ́e. Greenhouse–Geisser Correction. In: Salkind N, ed. *Encyclopedia of Research Design*. SAGE Publications, Inc.; 2010. doi:10.4135/9781412961288.n168
